# Consequences and Control of Multiscale Order/Disorder in Chiral Magnetic Textures

**DOI:** 10.1021/acsnano.3c04203

**Published:** 2023-10-06

**Authors:** Berit H. Goodge, Oscar Gonzalez, Lilia S. Xie, D. Kwabena Bediako

**Affiliations:** † Department of Chemistry, University of California, Berkeley, California 94720, United States; ‡ Max Planck Institute for Chemical Physics of Solids, 01187 Dresden, Germany; § Chemical Sciences Division, Lawrence Berkeley National Laboratory, Berkeley, California 94720, United States

**Keywords:** chiral soliton lattice, intercalated transition metal dichalcogenides, helimagnet, scanning transmission electron microscopy, Lorentz TEM

## Abstract

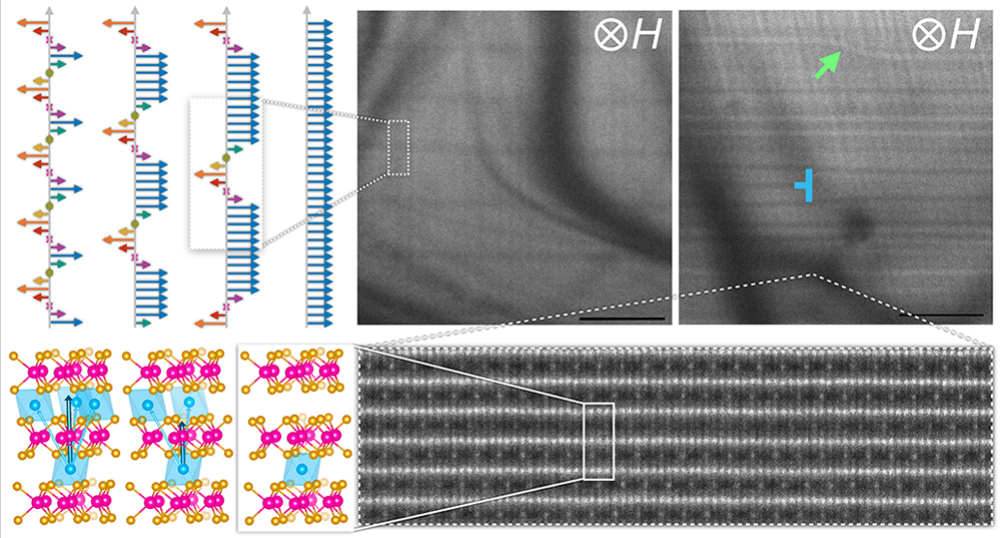

Transition metal intercalated transition metal dichalcogenides (TMDs) are promising platforms for next-generation spintronic devices based on their wide range of electronic and magnetic phases, which can be tuned by varying the host lattice or intercalant’s identity, stoichiometry, or spatial order. Some of these compounds host a chiral magnetic phase in which the helical winding of magnetic moments propagates along a high-symmetry crystalline axis. Previous studies have demonstrated that variation in intercalant concentrations can have a dramatic effect on the formation of chiral domains and ensemble magnetic properties. However, a systematic and comprehensive study of how atomic-scale order and disorder impact these chiral magnetic textures is so far lacking. Here, we leverage a combination of imaging modes in the (scanning) transmission electron microscope (S/TEM) to directly probe (dis)order across multiple length scales and show how subtle changes in the atomic lattice can tune the mesoscale spin textures and bulk magnetic response in Cr_1/3_NbS_2_, with direct implications for the fundamental understanding and technological implementation of such compounds.

Future electronic devices may rely on the manipulation of spin for information storage, mandating the exploration of solid-state platforms that enable magnetic order to be finely tuned and controlled.^1−4^ The potential benefits of miniaturization in terms of storage density and/or power efficiency may be realized either through the design of magnetic materials in which the atomic lattice imposes nanoscale confinement (that is, low-dimensional magnetic materials)^5,6^ or by exploiting atomic lattices which–even in bulk three-dimensional materials–produce nanoscale spin textures owing to a balance of disparate spin–spin correlations.^7−9^ Transition metal intercalated transition metal dichalcogenides (TMDs) offer a rich platform to investigate a wide range of magnetic phenomena.^10−22^ These materials can be described by the general chemical formula T_*x*_MCh_2_, where T and M are transition metals, Ch is a chalcogen, and *x* < 1. The intercalant stoichiometry *x* can direct the formation of superlattices through long-range ordering of the intercalant ions. These intercalation-derived superlattice structures in turn alter the overall symmetry of the crystal and dictate the bulk magnetic properties.^23^ For example, first-row transition metal intercalants have been found to result in 2*a*_0_ × 2*a*_0_ (principally at *x* = 1/4) or  (principally at *x* = 1/3) superlattices when the intercalants occupy the pseudo-octahedral sites between MCh_2_ layers, where *a*_0_ is the in-plane lattice constant of the nonintercalated MCh_2_ host lattice.^23^ The  superlattice in particular introduces noncentrosymmetry and chirality into the structure, giving rise to antisymmetric exchange interactions (also referred to as Dzyaloshinskii–Moriya, or DM, interactions)^24,25^ which compete with ferromagnetic exchange between adjacent planes and result in progressive rotation of spin orientation along the helical axis.^23,26^

Figure 1 provides an introductory summary of helical magnetic textures in intercalated TMDs, as well as their structural origins and experimental signatures. The intercalation of Cr into niobium and tantalum disulfides to produce Cr_1/3_NbS_2_ and Cr_1/3_TaS_2_ compounds leads to structures that possess the  superlattice, as depicted in Figure 1a and Supplemental Figure S1.^27^ In Figure 1b, the ordered arrangement of Cr intercalants between 2*H*-TaS_2_ layers is directly observed through atomic-resolution high-angle annular dark-field scanning transmission electron microscopy (HAADF-STEM) where the image contrast depends on elastic scattering of primary electrons in the STEM probe such that heavier nuclei give rise to brighter contrast. This electron micrograph shows how the Cr sublattices in each intercalant layer are offset from those in the adjacent layers such that the intercalants do not occupy the interstitial sites directly above or below each other.

**Figure 1:**
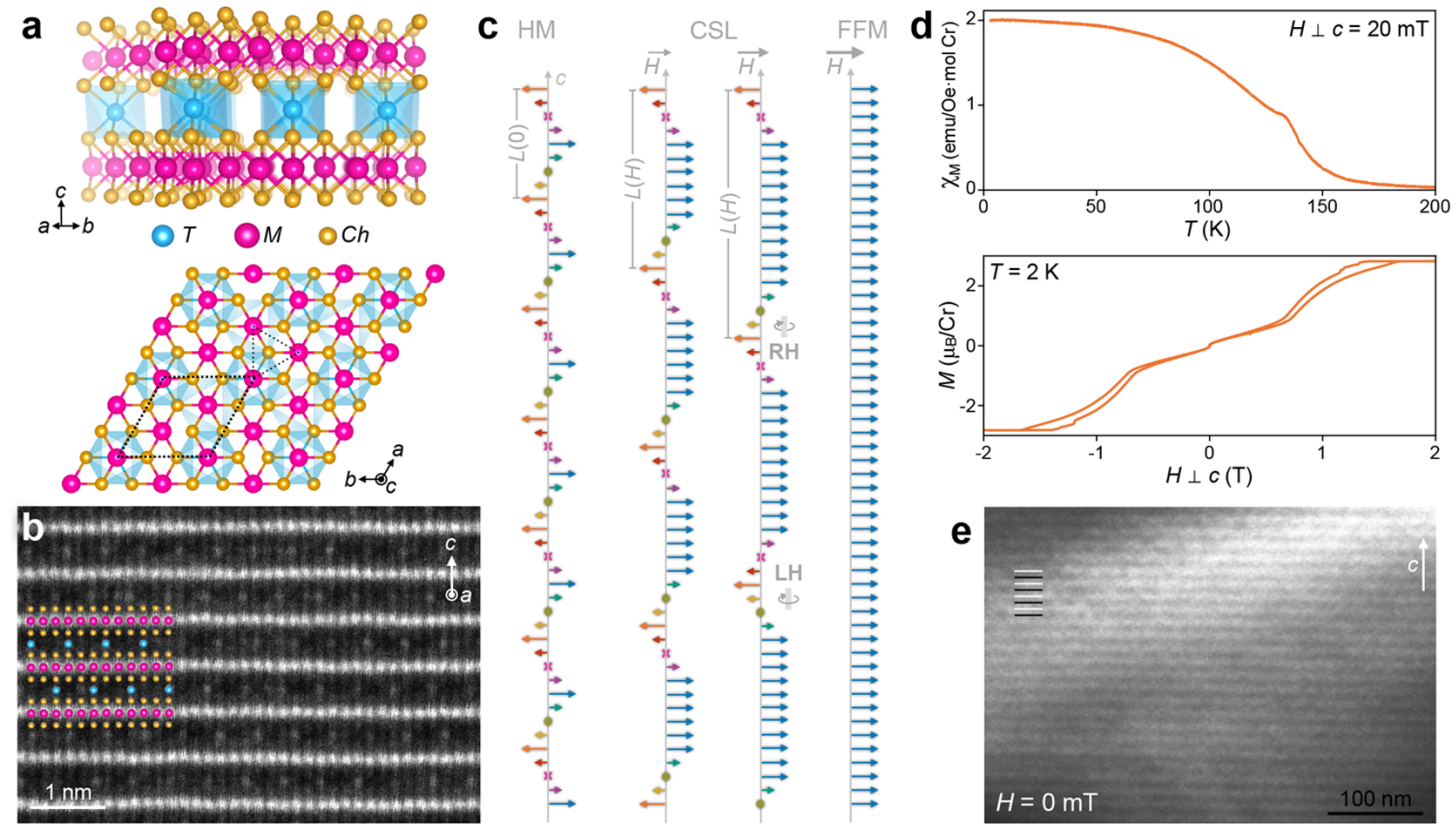
Tunable chiral magnetic textures in intercalated transition metal dichalcogenide (TMD) compounds. (a) Two views of the crystal structure of T_1/3_MCh_2_ where T is the intercalant, M is the metal ion, and Ch is the chalcogen. The dashed parallelogram indicates the  supercell where *a*_0_ is the lattice constant of the host MCh_2_ lattice. (b) Atomic-resolution HAADF-STEM image along the [100] projection and schematic overlay of Cr_1/3_TaS_2_, (Cr = cyan, Ta = magenta, S = gold). (c) Illustration of magnetic order along the crystallographic *c*-axis with spins in each plane oriented uniformly but with an angle offset with respect to adjacent layers. The zero-field helimagnet (HM) phase has a characteristic period *L*(0). With increasing applied fields, *H⃗*, the magnetic spiral begins to “unwind”, leading to chiral soliton lattice (CSL) phases with increasing periodicities, *L*(*H*), and, eventually, to a forced ferromagnetic (FFM) state. Soliton walls can follow left- or right-handed winding, as pictured in the second CSL phase. (d) Representative bulk magnetic measurements of Cr_1/3_TaS_2_, showing the magnetic susceptibility, χ_M_, as a function of temperature, *T* (top), and the magnetization, *M*, as a function of magnetic field, *H* (bottom). The field was applied in the *ab*-plane. (e) Zero-field cryo-LTEM (*T* ∼ 100 K) image showing the chiral helimagnetic order in Cr_1/3_TaS_2_, with a *L*(0) ∼ 17 nm. The slowly varying background is due to diffraction contrast from subtle bending in the thin lamella.

The broken inversion symmetry that arises from these intercalant positions and attendant DM interactions result in chiral helimagnetic (HM) phases^11,23,26,28,29^ that evolve from a zero-field texture comprising layers of aligned spins rotated relative to each other along the direction of the helical winding, which in these materials is the crystallographic *c*-axis (Figure 1c). Under an external magnetic field applied in the easy (*ab*) plane, these chiral textures begin to “unwind” such that regions of locally aligned spins are periodically separated by magnetic solitons, forming what is known as a chiral soliton lattice (CSL).^11,26,28,30,31^ Above a critical field strength, *H*_c_, all the spins align along the direction of the external field in a so-called forced ferromagnetic (FFM) state.^30^

This magnetic evolution can be observed in measurements of magnetic susceptibility as a function of temperature and magnetization as a function of applied magnetic field, which express a kink around the Curie temperature, *T*_C_, and a small hysteresis around *H*_c_, respectively (Figure 1d), both of which have been attributed to CSL formation and evolution.^28,30^ In addition, the HM texture can be directly imaged with Lorentz transmission electron microscopy (LTEM) when the sample is cooled *in situ* below its magnetic ordering temperature (here to *T* ∼ 100 K), as shown in Figure 1e for the zero-field HM phase in Cr_1/3_TaS_2_. In this measurement, the deflection of high-energy primary electrons in the TEM beam due to local moments in the sample creates dark and light contrast in over- and under-focus imaging conditions (Supplemental Figure S2).^11,26,32,33^ Consequently, in Figure 1e, the periodic horizontal stripes of bright and dark lines arise from the one-dimensional winding magnetic order revealing a 17 nm HM periodicity, *L*(0), in Cr_1/3_TaS_2_ consistent with previous reports.^34^ Qualitatively, the measurements in Figure 1d,e are considered to be common signatures of HM/CSL ordering. Quantitatively, the critical temperatures and fields as well as the real-space periodicity vary between different compounds depending on the relative strengths of exchange parameters, as will be discussed later.

Previous work within the family of intercalated TMD compounds has investigated mesoscale evolution of magnetic order^11,34,35^ and the subtle dependence of bulk magnetization response on a variety of tuning knobs, including stoichiometry,^36−38^ mechanical strain,^39^ and sample geometry.^40^ So far, however, a comprehensive study uniting structural and magnetic characterization across length scales from the atomic scale to microscale has been lacking. Such an analysis is critical for these materials because, as intercalation compounds, they are distinctively susceptible and sensitive to disorder on the intercalant (magnetic) sublattice, with severe consequences for the emergent magnetic properties.^15,18,19,23,41^

Here, we probe the structure and consequent magnetic behavior of HM systems, with a primary focus on Cr-intercalated 2*H*-NbS_2_, Cr_1/3_NbS_2_, which has thus far been more widely studied than the Cr_1/3_TaS_2_ counterpart introduced in Figure 1. We interrogate the effects of atomic order and disorder on mesoscopic magnetic textures and bulk properties of the emergent HM phases and CSL transitions. We show that subtle, local stoichiometric variations can lead to pronounced (up to 3-fold) changes to the CSL periodicity. Atomic-resolution electron microscopy unveils how these quantitative distinctions arise from different mechanisms for accommodating minimal extents of Cr deficiency, which correlate to the rate at which crystals are cooled during synthesis. Beyond magnetic periodicity, we find that concentrations of disorder in the atomic lattice additionally nucleate mesoscopic defects in the magnetic lattice, including dislocations, shearing, and heterochirality. Our study reveals the propagating impact of local and global order and disorder in intercalated TMD compounds and identifies key parameters for engineering high-quality, predictable materials for future fundamental studies and technological applications.

## Results and Discussion

### Structure and Chiral Helimagnetic Texture in Cr_1/3_NbS_2_

Single crystals of Cr_1/3_NbS_2_ were synthesized from the elements by chemical vapor transport at growth temperatures of 850–1000 °C, as detailed in the Methods. Two batches of crystals, differing principally in the cooling rate, were grown: one batch was cooled from the growth temperature at a rate of 20 °C/h, whereas the second batch was cooled more rapidly at 60 °C/h. Crucially, notwithstanding these synthetic variations, both batches of Cr_1/3_NbS_2_ samples are stoichiometrically equivalent within the error of our analysis (Supplemental Figures S3 and S4, Supplemental Table S1), suggesting that the Cr filling should vary by less than 1% between the two crystals.

**Figure 2:**
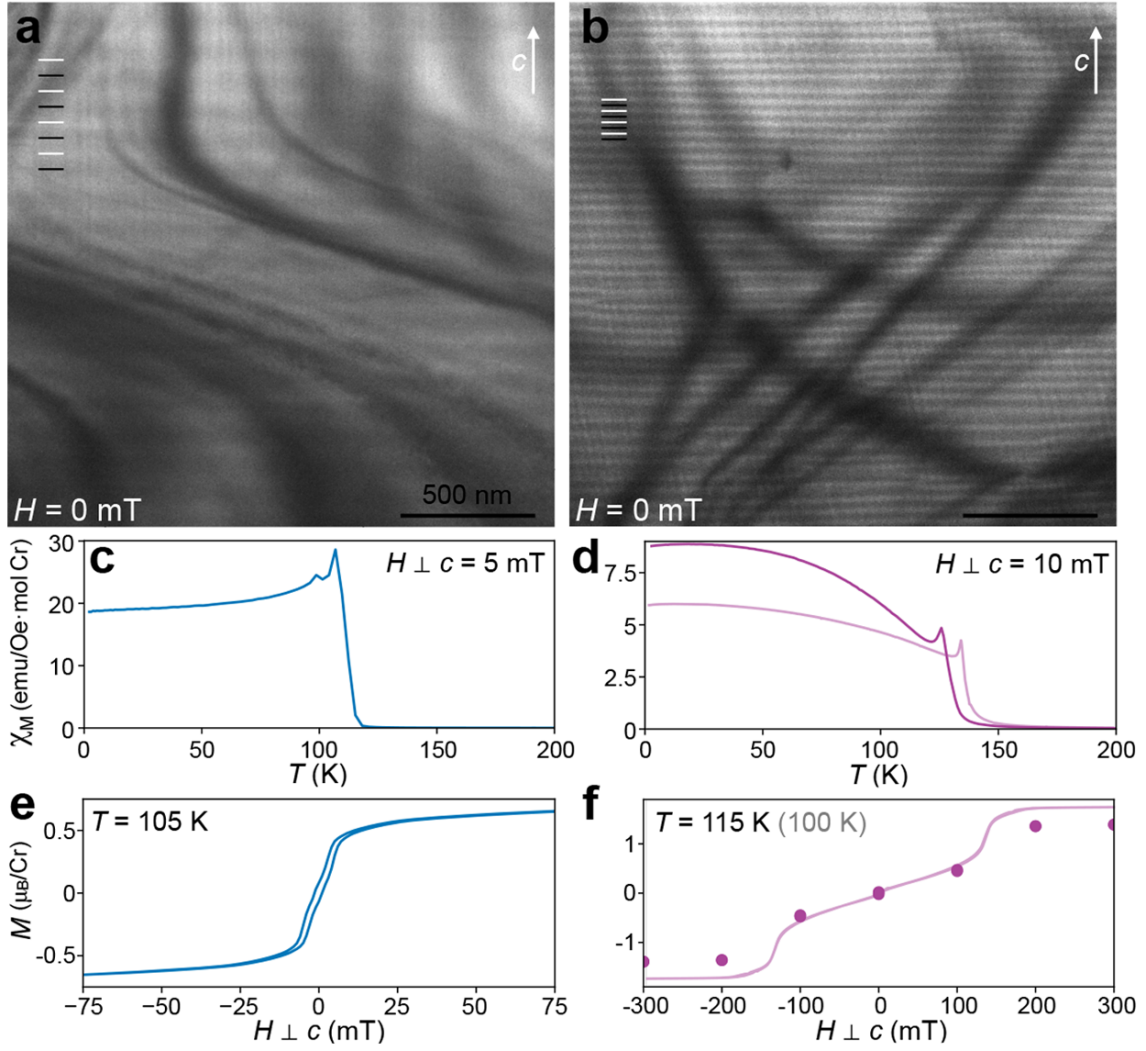
Varying magnetic order in two Cr_1/3_NbS_2_. (a, b) Zero-field cryo-LTEM (*T* ∼ 100 K) images showing chiral helimagnetic ordering in Cr_1/3_NbS_2_ samples with (a) HM *L*(0) ∼ 113 nm and (b) HM *L*(0) ∼ 43 nm. The curved bands of intensity variation are due to the diffraction contrast from subtle bending in the thin lamellae. (c, d) Magnetic susceptibility (χ_M_) as a function of temperature, *T*, for the two Cr_1/3_NbS_2_ samples in panels a and b, respectively. (e, f) Magnetization, *M*, as a function of magnetic field, *H*, for the two Cr_1/3_NbS_2_ samples in panels a and b, respectively, near the temperature of the cryo-LTEM measurements. Due to the coarse field sampling in panel f, we also include light traces in panels d and e showing comparable data from a second Cr_1/3_NbS_2_ sample from the same batch as that in panel b. Additional magnetization data for these and other crystals from the different synthesis batches are provided in Supplemental Figures S5 and S6. The external field was applied in the *ab*-plane. The static field or temperature of each measurement in panels c–f is provided in each.

Figure 2a,b shows cryo-LTEM images of cross-sectional TEM samples from these batches, revealing dramatically different HM periodicities: the slower-cooled sample exhibits a HM periodicity of ∼113 nm (Figure 2a) and the more rapidly cooled crystal possesses a HM periodicity of ∼43 nm (Figure 2b). We emphasize that the cooling discussed here refers to the cooling process during sample synthesis rather than the cooling rate of the TEM lamellae for our cryo-LTEM measurements, which are identical for all samples (see Methods). These periodicities are respectively longer than and in good agreement with previous reports in the same compound.^11,40^ For consistency, we refer to these as the “long-period” and “short-period” samples for the rest of our discussion. The single crystals from which these TEM samples were excised exhibit qualitatively similar magnetization responses with the characteristic CSL peak near *T*_C_ in the χ_M_(*T*) response, while the *M*(*H*) behavior exhibits a low field linear region with a sharp increase in *M* followed by saturation at *H*_c_, and narrow hysteresis (Figure 2c,d). Multiple crystals from each batch exhibit consistent behavior (Supplemental Figures S5 and S6), demonstrating systematic differences between the two cooling rates. Quantitatively, however, the critical fields differ by a factor of nearly 4: the long-period sample saturates near 50 mT while the short-period sample saturates at approximately 200 mT. We note that the temperature of the sample in our liquid nitrogen cryo-TEM holder is ∼100 K, near the kink in susceptibility which indicates a magnetic phase transition in the long-period Cr_1/3_NbS_2_. Although working near the magnetic phase transition could conceivably contribute additional fluctuations in the magnetic structures during our measurements, we do not observe any variations in the magnetic texture over the course of many hours during the experiment, including throughout normal temperature variations on the scale of several K, confirming that our experiments are sufficiently beyond the magnetic phase transition.

One distinction between the two Cr_1/3_NbS_2_ samples in the bulk magnetic properties is the low-temperature susceptibility. Most reports of Cr_1/3_NbS_2_ and related compounds show susceptibility responses which echo that in our short-period sample shown in Figure 2d, where the sharp peak near *T*_C_ is followed by increasing susceptibility to lower temperatures, similar to the traditional ferromagnetic response. Our long-period sample exhibits instead a flattening or slight decrease in susceptibility as the temperature is lowered. The origin for this distinct low-temperature behavior is as yet unknown, but similar results have been reported elsewhere in Cr_1/3_NbS_2_ compounds.^37,42^ Theoretical treatment of other helimagnetic systems suggests that there may be some dependence on the competition between the relevant exchange interactions,^43,44^ which will be discussed later. Future experimental investigations of any temperature dependence of the magnetic ordering below our current cryogenic baseline may also be insightful for understanding this divergence. Here, we focus on temperatures slightly below *T*_C_ where the HM phase is clearly observed by cryo-LTEM in both samples.

**Figure 3:**
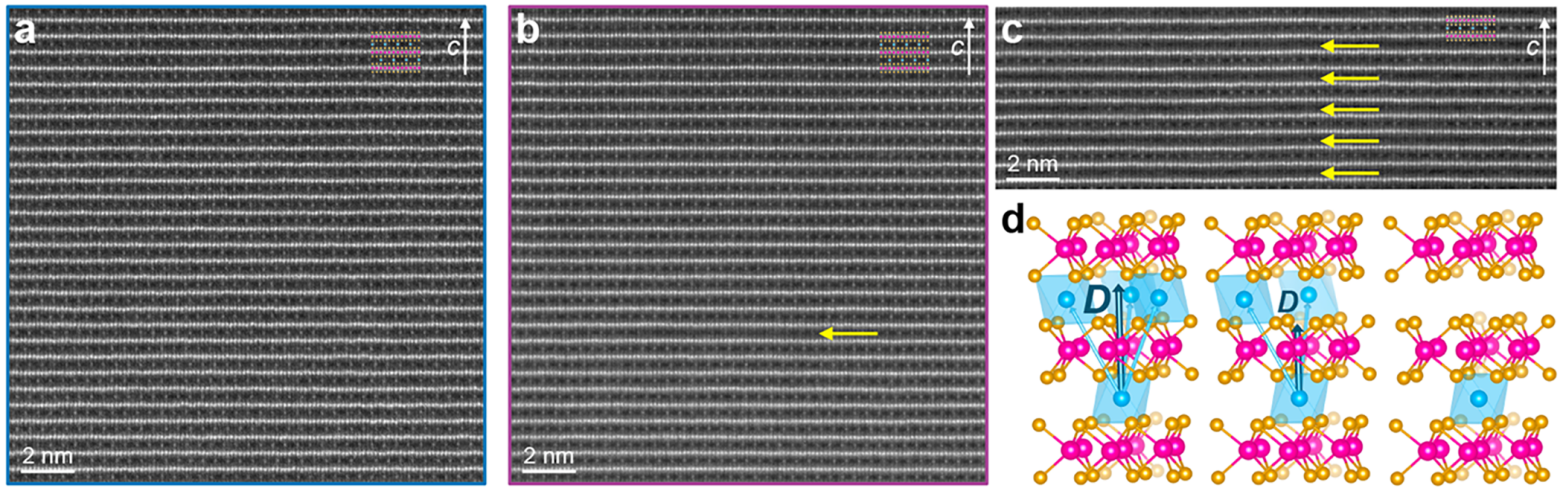
Atomic-scale insights to global vs local distribution of Cr vacancies. (a, b) Atomic-resolution HAADF-STEM images of the Cr_1/3_NbS_2_ samples in Figure 2a,b, respectively, with model overlays as shown in Figure 1. A local cluster of Cr vacancies is visible in panel b, marked with a yellow arrow. (c) HAADF-STEM image of a Cr-deficient (Cr_0.28_NbS_2_) sample prepared in the same conditions as that in panel b showing a high density of Cr vacancies similarly clustered. (d) Schematic representation of the impact of Cr vacancies on the antisymmetric DM interaction vector *D⃗* (dark teal arrow) based on addition of interlayer Cr–Cr couplings (cyan arrows) according to Moriya’s rules.

To investigate the origins of the dramatic differences in HM texture of the long- and short-period samples, we leverage atomic-resolution HAADF-STEM imaging (Figure 3a,b). Both samples show overall high crystallinity with no observable stacking faults or other defects in the parent TMD lattice and have a mostly uniform, well-ordered distribution of the Cr intercalants consistent with the model in Figure 1a. The short-period sample, however, exhibits local gaps in the intercalant lattice, as marked by a yellow arrow in Figure 3b. This lack of atomic contrast within the indicated few-nanometer region indicates a local absence of Cr intercalants or, equivalently, a local “pocket” of concentrated Cr vacancies. Similar vacancy clustering is also found in more strongly deficient Cr_0.28_NbS_2_ (Figure 3c) in which we find no signs of HM/CSL ordering (Supplemental Figures S8 and S7) as well as the related Fe-intercalated NbS_2_ compound^19^ and is reminiscent of the staging behavior that is common to graphite intercalation compounds.^45−47^ We found no such pockets within the long-period sample.

As discussed above, our compositional analysis indicates that both the long- and short-period Cr_1/3_NbS_2_ samples are stoichiometrically equivalent, or nearly so. Accordingly, let us assume that both samples are equally very slightly Cr-deficient such that comparable amounts of Cr vacancies are present in both samples. In the short-period sample, we observed that these vacancies are clustered in the pockets described above. Having observed no such pockets in the long-period sample, we posit that an equivalent number of vacancies that are clustered as pockets in the short-period sample are instead distributed randomly and uniformly throughout the long-period sample. The projection nature of HAADF-STEM imaging collapses the small but finite thickness of the TEM lamella into a single two-dimensional image, such that single vacancies within a column of occupied intercalant sites are effectively undetectable in the measurements here. We ascribe this difference in vacancy distribution to the rate at which the crystals were cooled from the elevated synthesis temperatures, 20 and 60 °C/h for the long- and short-period samples, respectively, surmising that slower cooling results in a dilute but globally homogeneous distribution of vacancies, while more rapid cooling results in pockets of concentrated vacancies.

We propose that this picture of Cr vacancy accommodation can further explain the vastly different magnetic periods. The winding of spins in the HM phase is dependent on the ratio of the DM vector between neighboring intercalants *i* and *j* in consecutive planes, *D⃗*_*ij*_, and their magnetic exchange coupling, *J⃗*_*ij*_. The wavevector of the winding period is given by *Q*_0_ = 2π[*L*(0)]^−1^ = arctan(*D*_*z*_/*J*),^11,48^ which for small values of *D*_*z*_/*J* can be approximated and rewritten as
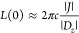
1where *L*(0) is the period in the (zero-field) HM phase, *c* is the lattice constant along the helical axis, *J* is the magnetic exchange coupling, and *D*_*z*_ is the magnitude of the DM interaction.^11,34,48^

Here, we can estimate a relative increase in the magnitude of *J*_*z*_/*D*_*z*_ based on the expanded *L*(0) of the long-period sample, suggesting that (*J*_*z*_/*D*_*z*_)_long_ ≈ 2.6 × (*J*_*z*_/*D*_*z*_)_short_. For compounds with similar lattice constants and identical intercalant species, little variation in magnetic exchange coupling *J*_*z*_ is expected, so for now we consider the magnetic pitch to be tuned primarily through the relative strength of *D*_*z*_,^34^ where a stronger DM interaction leads to larger azimuthal misorientation between interlayer spins and a shorter period. One factor that determines *D⃗*_*ij*_ is spin–orbit coupling (SOC). In the context of T_*x*_MCh_2_ systems, host lattices of heavier atoms possess stronger DM interactions,^25^ as reflected in the smaller HM period of Cr_1/3_TaS_2_ (Figure 1e) than in either sample of Cr_1/3_NbS_2_. Here, however, we observe a large apparent change in *J*_*z*_/*D*_*z*_ in two samples comprised of the same host lattice, that is, the same SOC and same *c*. Given the overall chemical and crystalline similarity between the two samples, we must thus consider more subtle possible mechanisms that may contribute to this discrepancy.

A schematic representation of the DM interactions that give rise to HM behavior is presented in Figure 3d, where couplings between a Cr ion in the bottom layer and three neighboring ions in the top layer are represented by cyan vectors between atomic sites. The sum of the corresponding *D⃗*_*ij*_(*i* ≠ *j*) vectors results in an overall *D⃗* which points along the crystalline *c*-axis. The primary difference that we observe between the short- and long-period Cr_1/3_NbS_2_ samples is the presence and lack of clustered Cr vacancy pockets, respectively. We propose a possible description of how Cr vacancy clusters can influence the strength of *D*_*z*_ as compared to a random distribution of single Cr vacancies. We posit that when several Cr ions are missing, as in the case of a vacancy cluster, the interlayer coupling is locally fully suppressed. On the other hand, when a single Cr ion is removed, as in the case of an isolated vacancy, the magnitude of the *D⃗* vector is decreased while the overall direction remains along the crystallographic *c*-axis when averaged over random vacancies across the whole sample. The strength of this effect (change in *J*_*z*_/*D*_*z*_ by ∼2.6 times) for such a seemingly subtle difference in the distribution of diffuse vacancies (again, we emphasize that both samples are measured to be within 1% of 1/3 stoichiometric Cr filling) points to an extremely sensitive dependence of the magnetic coupling in these materials on dilute interactions.^49^

We propose an understanding of the helimagnetic ordering in the short- and long-period samples by considering the localized versus distributed nature of Cr vacancies (naively ignoring, for now, the impact of intercalant disorder on the exchange *J*_*z*_ and considering only explicit change in *D*_*z*_). The long-period sample consists of random Cr vacancies at a global scale which reduces the overall strength of *D*_*z*_, while the short-period sample consists of locally clustered Cr vacancies such that the global magnitude of *D*_*z*_ remains relatively large, except at the local vacancy clusters, and the global HM period is correspondingly shorter. A more comprehensive theoretical understanding of these different types of disorder will take into account the associated impact on exchange coupling *J⃗* in addition to the DM interaction.^49^ A similar concept of correlated disorder in the Cr sublattice has been previously proposed to explain variations in reported *T*_C_ throughout the literature based on diffuse scattering measurements and Monte Carlo simulations,^41^ but the precise atomic-scale structure of the Cr disorder has not been directly observed until now. Further confirmation of this picture may be possible with a unification of advanced techniques such as quantitative magnetic imaging with high spatial resolution^50,51^ combined with atomic vacancy identification by ptychographic reconstruction techniques.^52^

It is further interesting to note differences in the characteristic intercalant (dis)order in various T_*x*_MCh_2_ compounds: in seemingly stoichiometric Fe_0.25_TaS_2_, for example, changes to synthetic conditions (e.g., cooling rate) manifest as large changes to the ferromagnetic coercivity and are attributed to variations in the population of 2*a*_0_ × 2*a*_0_ and  superlattices.^16^ On the other hand, here (and indeed, even in the more strongly deficient Cr_0.28_NbS_2_ shown in Figure 3c) we find no signatures of mixed 2*a*_0_ × 2*a*_0_ ordering in addition to the . Future studies that consider systematic variation of intercalant species, stoichiometry, and host lattice may uncover if key ingredients (such as certain magnetic behavior) favor specific structural motifs.

### Evolution of Chiral Spin Textures in External Magnetic Fields

The technological promise of these and other magnetically textured materials lies not just in controlling their static ordering but in dynamically and predictably tuning magnetic order with an external stimulus. It is therefore imperative to additionally understand the evolution of magnetic textures under an applied field. Here, we find that the two Cr_1/3_NbS_2_ samples undergo notably different CSL phase evolution, which further emphasizes the impact of local versus global disorder in these systems.

**Figure 4:**
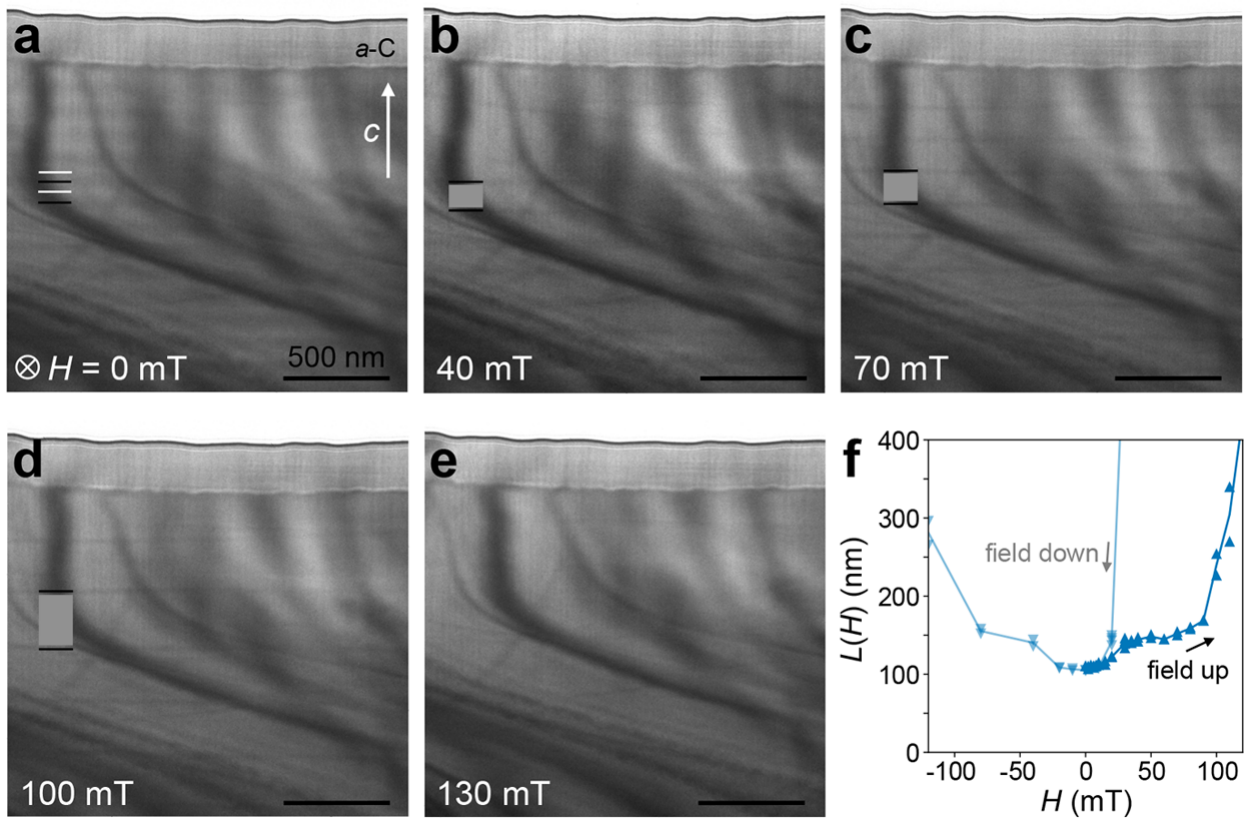
Evolution of magnetic texture in the long-period Cr_1/3_NbS_2_ sample. (a–e) Cryo-LTEM (*T* ∼ 100 K) images of the chiral magnetic texture in the long-period Cr_1/3_NbS_2_ sample under applied magnetic field orthogonal to the *c*-axis with field strengths indicated for each frame. Light and dark lines in panel a are added to highlight the peaks and troughs of the periodic HM contrast. In panels b–d, the dark lines denote soliton walls, and the medium gray boxes mark regions of local FM ordering in the CSL phase. (f) Progression of the soliton period *L*(*H*) tracked by *in situ* LTEM imaging under increasing (dark, upward-pointing triangles) and decreasing (light, downward-pointing triangles) magnetic fields. A layer of protective amorphous carbon (*a*-C) from the specimen preparation is visible along the top of the sample; the top of each image is vacuum.

Figure 4 shows the evolution of the CSL phase in the long-period Cr_1/3_NbS_2_ sample under an *in situ* applied magnetic field. The soliton walls separating locally ferromagnetic domains as the helimagnetic order begins to unwind appear in LTEM as either dark or bright lines (Supplemental Figure S9).^11,35^ Here, all of the solitons show the same left-handed chirality throughout the CSL phase evolution in a given field direction (i.e., positive *H*), as indicated by consistent dark contrast in the underfocused Fresnel cryo-LTEM conditions. The soliton contrast inverts when the applied field is reversed (negative *H*), further consistent with homochirality throughout the sample and under both field directions (Supplemental Figure S10).

As expected, the soliton lattice spacing increases with the field strength until no solitons remain visible, and the sample can be considered in the FFM phase above ∼130 mT (Figure 4e). The external field at which no soliton walls are visible by cryo-LTEM is somewhat larger than the critical field *H*_c_ at which the magnetization *M* appears to saturate (Figure 2c): ∼136 mT versus ∼25–50 mT, respectively. Quantitative comparisons between these measurements are complicated by the geometric differences between the bulk crystals and thin TEM lamellae, the distinct boundary conditions of which can impact the demagnetization fields and pinning effects in each case. Another possible contribution to this apparent discrepancy may arise from the relative sensitivities of the two measurements to very low densities of soliton walls: real-space imaging by cryo-LTEM can clearly resolve single solitons whenever the spacing is less than the dimension of the prepared lamella, here ∼3–4 μm. The magnetic volume fraction, however, contributed by solitons becomes vanishingly small at very large *L*(*H*) such that the bulk magnetic response may appear effectively saturated even if some small density of soliton walls remain. To our knowledge, similarly correlated measurements by bulk and real-space techniques under applied fields have not been reported for other crystals, but reports of critical fields measured by LTEM^35^ and bulk magnetization^26^ on samples from the same group show a similar qualitative trend.

The field-dependent CSL period *L*(*H*) increases in discrete steps with abrupt changes, as has been observed previously^11,35^ (Figure 4f and Supplemental Video 1). We also observe a large hysteresis in *L*(*H*) when the external field is ramped down,^35^ showing a much more abrupt transition out of the FFM phase with a sudden onset of soliton walls (Supplemental Figure S11). In an increasing field, the value of *L*(*H*) shows some tendency to favor certain “metastable” lengths, for example, remaining relatively stable near 150 nm for *H* between 30 and 80 mT (Figure 4f). We note that the CSL lengths within this plateau appear to coincide with the approximate thickness of our TEM specimen (see Supplemental Figure S12) and speculate that this may point to the relevance of some dimensional confinement or other boundary conditions as has been recently discussed in the context of fabricated nanowedges of the same material.^40^ Future efforts to explore these effects more systematically with both experimental measurements and theoretical models should provide more insight in this regard. Overall, however, the magnetic phases in the long-period sample are found to be fairly well-ordered and reproducible.

**Figure 5:**
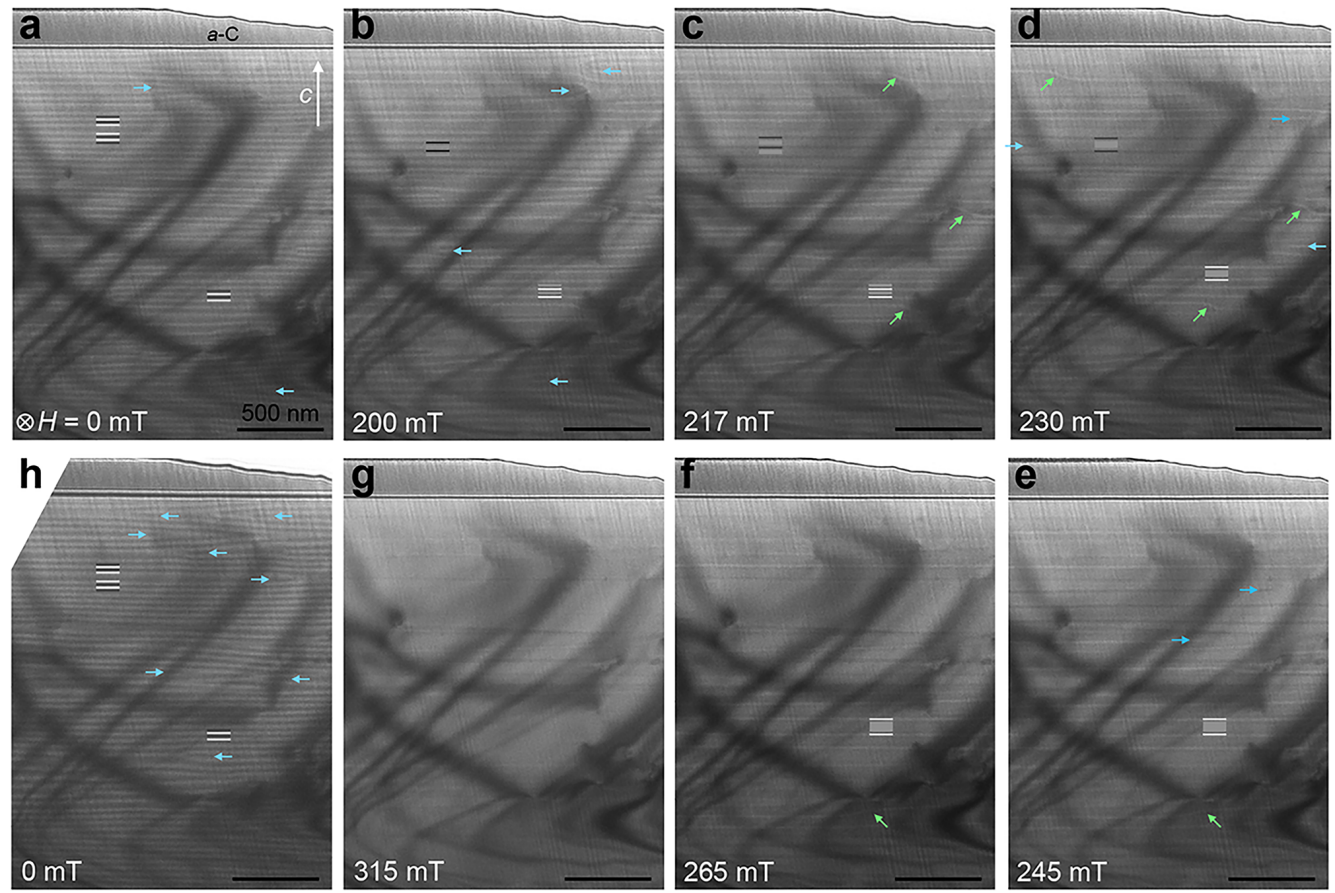
Evolution of magnetic texture in short-period Cr_1/3_NbS_2_. (a–g) Cryo-LTEM (*T* ∼ 100 K) images of the chiral magnetic texture in the short-period Cr_1/3_NbS_2_ sample under increasing applied magnetic fields orthogonal to the *c*-axis, as indicated for each frame. Light and dark lines in panel a are added to highlight the peaks and troughs of periodic HM contrast. In panels b–f, the dark and bright lines denote soliton walls of opposite chirality, and the medium gray lines mark regions of local FM ordering in the CSL phase. (h) Magnetic texture was restored when the external field was removed following the ramp to 315 mT in panel g. Dislocations and shearing in the magnetic lattice are highlighted with blue and green arrows, respectively. A layer of protective amorphous carbon (*a*-C) from the specimen preparation is visible along the top of each micrograph; the top right corner in each image is vacuum.

**Figure 6:**
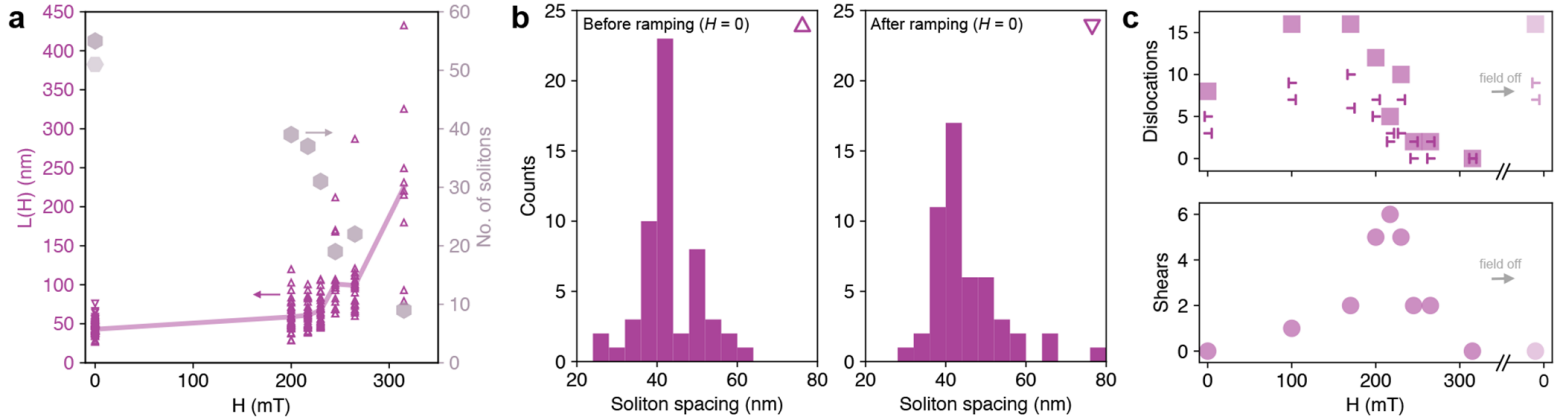
Quantification of soliton spacing/density and defects in short-period Cr_1/3_NbS_2_. (a) Measured soliton spacing *L*(*H*) (left axis) and number of solitons within a 4.4 μm range (right axis) for applied external fields. Each *L*(*H*) data point is the distance between two neighboring soliton walls. A line connecting the average spacing at each field is added as a guide for the eye. Measurements at *H* = 0 before and after the field sweep are marked by upward (△) and downward (▽) symbols, respectively (note that the data points significantly overlap as displayed). (b) Distribution of soliton spacing at *H* = 0 before and after the field sweep is replotted from panel a as histograms. (c) Counts of dislocations and shear defects extracted from the same cryo-LTEM image field series. Dislocations are distinguished by opposite sign (bar with line to right or left) and combined into a total count (□). Shear defects are considered regions in a soliton with notable curvature which are not obviously due to accommodating a dislocation.

In contrast, the magnetic texture of the short-period sample exhibits considerably different behavior (Supplemental Video 2). Figure 5 contains a similar *in situ* magnetic field cryo-LTEM image series through the CSL phase. Unlike the long-period Cr_1/3_NbS_2_, which progresses through uniform *L*(*H*) at different fields, the short-period Cr_1/3_NbS_2_ exhibits widely varied soliton spacings at every field such that a single *L*(*H*) cannot be reasonably defined. Still, with increasing field, the soliton spacings increase and the total number of solitons within a given sample area decreases, as quantified in Figure 6a, where soliton spacing and density are extracted from a vertical profile spanning 4.4 μm through the center of each image in Figure 5. This variation may reflect additional inhomogeneity in Cr vacancies between the two samples, as local concentrations of Cr vacancies would have an analogous local impact on *D⃗*_*ij*_, and should not significantly alter the magnitude of *D⃗*_*ij*_ throughout the bulk of the crystal. Figure 5 also reveals that the soliton lattice of the short-period crystal contains various “defects” in the spin texture.^53^ These include shearing, where solitons are observed to bend across tens of nanometers along *c* (marked in Figure 5 with green arrows), and dislocations, where soliton walls suddenly terminate in the *ab* plane (marked in Figure 5 with blue arrows).

Based on the atomic resolution HAADF-STEM imaging in Figure 3, we propose that these spin texture defects of shearing and dislocations, which are observed in the short-period sample (but are notably absent in the long-period sample), may arise or nucleate at regions of clustered Cr vacancies that form corresponding defects in the intercalant (magnetic) sublattice. Moreover, the presence of both dark and light stripes in Figure 5b–e reveals a mix of left- and right-handed solitons that appear with opposite contrast under identical underfocused imaging conditions (Supplemental Figure S10), demonstrating heterochirality of magnetic order in the short-period sample, which is not observed in the long-period sample (Supplemental Figure S13). Although we find the overall behaviors of both short- and long-period samples quite reproducible, we do observe some stochastic variation in terms of the precise location and density of defects within the magnetic order and the local distribution of CSL spacing, calling for further systematic and statistical analysis of the origin and evolution of these textures in future studies.

These stark differences between the (in)homogeneity of the magnetic order between the short- and long-period samples are again consistent with their relative cooling rates after crystal growth. The chirality of the magnetic texture is defined by the inversion symmetry breaking in the Cr sublattice.^34^ It has been shown previously that more rapid cooling through the high-temperature chiral structural transition leads to a higher density of topological defects in Cr_1/3_TaS_2_.^34^ Here, we propose an atomic-scale origin for these observations, whereby faster cooling rates result in pockets of locally fully occupied and ideally ordered lattices with strong DM interactions within given helical magnetic domains but more global lattice disorder, which in turn give rise to heterochirality in the magnetic lattice and CSL winding. Slower cooling rates result in more homogeneously distributed disorder/vacancies which uniformly weaken DM interactions and lead to a low density of helical domains but preserve homochiral helimagnetism. Left- and right-handed atomic domains are indistinguishable by HAADF-STEM imaging (Supplemental Figure S14), but future measurements directly correlating the atomic-scale ordering and corresponding local magnetic chirality may be possible with advanced techniques.

At 315 mT (the largest field reachable by our LTEM experiments), the sample appears nearly transitioned to the FFM phase, although some solitons remain visible (Figure 5f). As with the long-period sample, the critical field *H*_c_ extracted from cryo-LTEM experiments (315 mT) is larger than that determined from the bulk magnetization plateau (∼200 mT). The remaining solitons separate regions of the sample that hosted either left- or right-handed textures, possibly pointing to “trapped” whole or fractional soliton windings at these domain boundaries,^35^ although the lattice-scale configuration of spin texture across one of these left-to-right-hand domain walls has not yet been quantitatively determined. The sizes of these single-handed domains likely also impact the discrete evolution of *L*(*H*) through soliton confinement effects.^35,48,54^

A detailed description of the evolution (including movement, creation, and annihilation) of magnetic defects remains an area of active study^40,53^ to which our results provide mesoscale insights. For example, many of the soliton dislocations and shear defects are observed to move laterally with the application of external field (see Supplemental Video 2), consistent with a picture of solitons “escaping” from the sample edges normal to the helical axis.^40,55^ Studies in other HM systems have suggested that soliton dislocations annihilate in pairs of opposite sign,^56^ examples of which we indeed observe here. We also find other instances, however, in which single soliton dislocations appear to “self-annihilate” without a complementary pair (Supplemental Figure S15). Similar to the long-period sample, the magnetic evolution in the short-period Cr_1/3_NbS_2_ also shows stepwise changes in local soliton spacing (see Supplemental Video 2) and significant hysteresis between upward or downward ramping of the field (Supplemental Figure S16). Indeed, this sample demonstrates that the transitions can be extremely abrupt, for example, showing a ∼5× change in local *L*(*H*) as the applied field is decreased within the window of 1 mT (Supplemental Figure S17). Paterson et al. compare similar observations to a supercooled transition out of the FFM phase and propose that the “hysteresis of disorder” (i.e., the magnetic lattice shows more disorder when the field is decreased from FFM into the CSL phase) also observed here may point to the metastability of soliton dislocations.^53^ The magnetic texture in our sample also has the additional complexity of shear defects, which are seemingly favored at intermediate fields when solitons are comparatively far apart, as illustrated by Figure 6c and may point to the relative energetic costs of different kinds of defects depending on the local magnetic environments. A mechanistic understanding of these and other dynamic effects will likely require a combination of experimental measurements cycling through several such phase transitions with additional phenomenological and computational analysis.

## Conclusion

The stark differences in the static zero-field magnetic order and the dynamic evolution of chiral magnetic textures in Cr_1/3_NbS_2_ emphasize both the promise of these materials as highly tunable magnetic systems and the pressing need to understand how nanoscale disorder in various manifestations impacts the meso- and bulk-scale properties. Our results show the large degree to which chiral helimagnetic textures in Cr_1/3_NbS_2_ (and likely extended to analogous materials such as Cr_1/3_TaS_2_ and Co_1/3_MS_2_ (M = Nb, Ta))^34,57,58^ depend strongly on the subtle variations in single crystal synthesis and the varied effects of structural lattice disorder within Cr_1/3_NbS_2_ on spin texture disorder and the coexistence of heterochirality. Our findings also shed light on, and may offer some explanations for, discrepancies within the literature, in which the properties of Cr_1/3_NbS_2_ vary across different studies.^11,55,59,60^ Future work should leverage additional approaches, including theoretical methods, to systematically and explicitly investigate how lattice structure, exchange interactions, and vacancy distribution—especially in such dilute limits—lead to the observed behaviors. Future experimental efforts should also explore additional dimensions of parameter space on the magnetic order, for example harnessing recent advances in continuously variable temperature cryogenic S/TEM sample holders^61^ to explore the impact of cooling rate or temperature cycling through the magnetic transition. Here, we begin to bridge the gap across these multiscale hierarchies by demonstrating how different distributions of Cr vacancies can lead to global changes in the magnetic properties. Understanding the connection between bulk magnetization measurements and the real-space evolution of the helical ordering in these Cr-intercalated materials and the atomic-scale origins of these behaviors will enable future engineering of functional devices based on this rich class of materials.

## Methods

### Single Crystal Growth

Single crystals of Cr_*x*_NbS_2_ and Cr_*x*_TaS_2_ were grown by using chemical vapor transport (Table 1). Powders of elemental Cr (−100 +325 mesh, 99.97%, Alfa Aesar), Nb (−325 mesh, 99.99% excluding Ta, Ta ≤ 500 ppm, Alfa Aesar), Ta (−100 mesh, 99.98% metal basis, Nb 50 ppm, Alfa Aesar), S (99.999%, Acros Organics), and I_2_ (99.999%, Spectrum Chemicals) were used as received.

**Table 1:** Synthetic Conditions Employed in the Synthesis of Cr_*x*_TaS_2_ and Cr_*x*_NbS_2_

sample	gradient (Δ, °C)	Cr/M/S	I_2_ concn (mg/cm^3^)	cooling rate (°C/h)
Cr_0.33_TaS_2_	100	0.47:1:2.1	2	20
Cr_0.33_NbS_2_ (long)	100	0.48:1:2.1	2	20
Cr_0.33_NbS_2_ (short)	200	0.35:1:2	1.3	60
Cr_0.28_NbS_2_	200	0.31:1:2	5	60

#### Growth of Cr_0.28_NbS_2_ Sample

Elemental Cr (47.2 mg, 0.31 equiv), Nb (270.4 mg, 1.00 equiv), and S (186.6 mg, 2.00 equiv) were sealed in a fused quartz ampule (14 mm inner diameter, 1 mm wall thickness, 48 cm long) under vacuum (approximately 1 × 10^–5^ Torr), along with 485.2 mg I_2_ (5 mg/cm^3^). The ampule was placed in a MTI OTF-1200X-II two-zone tube furnace with the hot zone maintained at 1050 °C and the cold (growth) zone maintained at 850 °C for 14 days, before cooling to room temperature at 60 °C/h. Plate-shaped crystals with a hexagonal habit of up to approximately 5 × 4 × 0.5 mm^3^ were obtained.

#### Growth of Long-Period Cr_1/3_NbS_2_ Sample

Elemental Cr (22.5 mg, 0.48 equiv), Nb (84.2 mg, 1.00 equiv), and S (60.9 mg, 2.1 equiv) were sealed in a fused quartz ampule (14 mm inner diameter, 1 mm wall thickness, 29 cm long) under vacuum (approximately 1 × 10^–5^ Torr), along with 90.1 mg of I_2_ (2 mg/cm^3^). The ampule was placed in a MTI OTF-1200X-II two-zone tube furnace with the hot zone maintained at 1100 °C and the cold (growth) zone maintained at 1000 °C for 14 days, before cooling to room temperature at 20 °C/h. Plate-shaped crystals with a hexagonal habit of up to approximately 2 × 2 × 0.5 mm^3^ were obtained.

#### Growth of Short-Period Cr_1/3_NbS_2_ Sample

Elemental Cr (25.7 mg, 0.35 equiv), Nb (131.9 mg, 1.00 equiv), and S (90.6 mg, 2.00 equiv) were sealed in a fused quartz ampule (14 mm inner diameter, 1 mm wall thickness, 48 cm long) under vacuum (approximately 1 × 10^–5^ Torr), along with 38.5 mg of I_2_ (1.3 mg/cm^3^). The ampule was placed in a MTI OTF-1200X-II two-zone tube furnace with the hot zone maintained at 1050 °C and the cold (growth) zone maintained at 850 °C for 14 days, before cooling to room temperature at 60 °C/h. Plate-shaped crystals with a hexagonal habit of up to approximately 4 × 4 × 0.5 mm^3^ were obtained^.^

#### Growth of Cr_1/3_TaS_2_

Elemental Cr (19 mg, 0.47 equiv), Ta (141 mg, 1.00 equiv), and S (53 mg, 2.1 equiv) were sealed in a fused quartz ampule (14 mm inner diameter, 1 mm wall thickness, 25 cm long) under vacuum (approximately 1 × 10^–5^ Torr) along with 76 mg of I_2_ (2 mg/cm^3^). The ampule was placed in a MTI OTF-1200X-II two-zone tube furnace, with the hot zone maintained at 1100 °C and the cold (growth) zone maintained at 1000 °C for 14 days, before cooling to room temperature at 20 °C/h. Plate-shaped crystals with a hexagonal habit of up to approximately 2 × 2 × 0.3 mm^3^ were obtained.

### Bulk Characterization

DC magnetization measurements were carried out on a Quantum Design Physical Property Measurement System, Dynacool, equipped with a 12 T magnet by using either the Vibrating Sample Magnetometer option or the AC Measurement System II option. Single crystals were affixed to quartz sample holders with GE Varnish such that the magnetic field was applied perpendicular to the crystallographic *c*-axis. Energy dispersive X-ray spectroscopy was acquired on an FEI Quanta 3D FEG or a Scios 2 DualBeam scanning electron microscope with an accelerating voltage of 20 kV.

### Electron Microscopy

We conducted measurements of two individual TEM lamellae cut from each crystal in our study (both the long- and short-period Cr_1/3_NbS_2_) and observe consistent behavior in each case (LTEM images of the second short-period lamella are included in Supplemental Figures S16 and S17). Cross-sectional S/TEM samples were prepared by the standard focused ion beam (FIB) lift-out method in Thermo Fisher Scientific Helios G4 UX or Scios 2 DualBeam. Samples were thinned to maximize the total area of electron transparency (*t*/λ ∼ 1–1.5 at 300 kV = ∼90–135 nm) for magnetic imaging (Supplemental Figure S12).

High-angle annular dark-field (HAADF)-STEM images were collected in the thinnest parts of the prepared lamellae, near the top edge, in regions that were significantly thinner (*t*/λ ∼ 0.3 at 300 kV = ∼30 nm). The intercalant (Cr) order was found to be extremely sensitive to the high-energy STEM probe, so HAADF-STEM imaging was performed on a Thermo Fischer Spectra 300 X-CEFG operating at 120 kV with low probe currents, <20 pA, convergence angle of 24 mrad, and inner (outer) collection angles of 68 (200) mrad. To minimize the possibility of introducing additional disorder in the intercalant lattice during measurement, all HAADF-STEM images were acquired from regions without prior exposure (other than limited doses at very low magnification for the purposes of sample alignment). For each image, a small (few-nm^2^) nearby region was used to focus the STEM probe before blanking the beam to navigate to a fresh region of the specimen and letting the stage stabilize for atomic-resolution image acquisition. In this way, large total areas of each sample were imaged with atomic resolution to build a statistical picture of the relative density of clustered Cr vacancies. STEM measurements were carried out prior to the LTEM experiments to ensure that the samples were imaged in as “pristine” condition as possible, and exposure was confined to only the very thinnest regions of the lamellae near the vacuum edge, such that the mesoscopic bulklike regions of the sample remain relatively pristine for our subsequent LTEM experiments.

Cryogenic Lorentz transmission electron microscopy (cryo-LTEM) was performed in an FEI Titan Themis instrument operating at 300 kV. A Gatan 636 side-entry double-tilt liquid nitrogen holder was used to cool the samples to ∼100 K. Both Cr_1/3_NbS_2_ samples were cooled simultaneously in the TEM to eliminate the possibility of additional variation in magnetic textures due to cooling rate during the experiment. We estimate that the samples reach cryogenic (*T* ∼ 100 K) temperatures from ambient (*T* ∼ 293 K) conditions within ∼0.5–1 h, but note the nonlinear cooling profile as the holder reaches its equilibrium temperature. Over-, under-, and in-focus LTEM images were collected on a Ceta CCD camera with 0.5–4 s acquisition times. Variable magnetic field was applied by adjusting the strength of the objective lens according to previous calibration of the system. To correct for the image shift and rotation caused by changing fields during *in situ* data collection, each frame in the LTEM series was realigned by rotation and rigid shift.

While in HAADF-STEM imaging the dose is primarily experienced as a highly focused probe exposed for a very short time which leads to a relatively high flux (dose rate per area) through the sample, in TEM the flux can be much lower as the sample is exposed to a parallel beam of electrons spread over large areas. Here, the L/TEM imaging was performed at cryogenic temperatures, which also reduces the effects of beam damage in many compounds. To ensure that exposure to the TEM beam did not induce changes in the magnetic order, we observed the magnetic texture under continuous exposure over the course of many minutes to confirm its stability. We further find the magnetic textures to be fully reversible and reproducible across multiple positive and negative sweeps of the applied field. The reproducibility of our observations and recoverability of the zero-field magnetic texture over many hours of measurement further demonstrate that the TEM exposure did not induce significant changes to the observations of magnetic order in our samples.

## Data Availability

The data contained within and relevant to the finding of this manuscript, including magnetization and magnetic susceptibility physical property measurements, scanning transmission electron microscopy images, and cryogenic Lorentz transmission electron microscopy images, have been deposited in the Platform for the Accelerated Realization, Analysis, and Discovery of Interface Materials (PARADIM) database (https://doi.org/10.34863/cwz8-5049). Bulk property data is provided in .csv format; S/TEM images are provided in .tif format. *In situ* magnetic field sweep LTEM series are provided in .gif format after image alignment. Raw .tif stacks are not posted due to large file size and can be obtained upon request to the authors.

## Supporting Information Available

Crystal models of T_*x*_MCh_2_ with *x* = 1/3; cryo-Lorentz TEM of Cr_1/3_TaS_2_; SEM-EDX spectra for long- and short-period Cr_1/3_NbS_2_ samples; comparison of SEM-EDX spectra for long- and short-period Cr_1/3_NbS_2_ samples; magnetization behavior of three slow-cooled Cr_1/3_NbS_2_ samples; magnetization behavior of four fast-cooled Cr_1/3_NbS_2_ samples; additional characterization of the deficient Cr_0.28_NbS_2_ sample; cryo-LTEM characterization of the deficient Cr_0.28_NbS_2_ sample; schematic diagram of LTEM image contrast; schematic diagram of LTEM image contrast under reversed fields; cryo-LTEM of long-period Cr_1/3_NbS_2_ with decreasing fields; thickness maps of Cr_1/3_NbS_2_ S/TEM lamellae; analysis of magnetic chirality in the long- and short-period Cr_1/3_NbS_2_ samples; atomic origins of chiral magnetic order; annihilation of soliton dislocations under applied field; cryo-LTEM of short-period Cr_1/3_NbS_2_ sample for fields ramping up and down; cryo-LTEM of short-period Cr_1/3_NbS_2_ showing abrupt onset of CSL order; and elemental concentrations measured by SEM-EDX (PDF)
Cryo-LTEM field ramp of long-period Cr_1/3_NbS_2_ (MOV)
Cryo-LTEM field ramp of short-period Cr_1/3_NbS_2_ (MP4)

## Supplementary Material

nn3c04203_si_001.pdf

nn3c04203_si_002.mov
