# Supplementary material for: Consequences and Control of Multiscale Order/Disorder in Chiral Magnetic Textures
Source: ACS Nano. 2023 Oct 6;17(20):19865–76. doi: 10.1021/acsnano.3c04203 (PMC10604074; doi:10.1021/acsnano.3c04203)
Supplement: Supplementary file 1 — nn3c04203_si_001.pdf [file nn3c04203_si_001.pdf]

*Supporting Information for*

# Consequences and control of multi-scale (dis)order in chiral magnetic textures

Berit H. Goodge[1,2,†], Oscar Gonzalez[1,†], Lilia S. Xie[1], and D. Kwabena Bediako[1,3,*]

[1]*Department of Chemistry, University of California, Berkeley, CA 94720, USA*
[2]*Max Planck Institute for Chemical Physics of Solids, 01187 Dresden, Germany*
[3]*Chemical Sciences Division, Lawrence Berkeley National Laboratory, Berkeley, CA 94720, USA*
[*]*Correspondence to: bediako@berkeley.edu*
[†]*These authors contributed equally to this work*

## List of Figures

S1 Crystal models of $T_xMCh_2$ with $x = 1/3$. . . . . . . . . . . . . . . . . . . . . . 1
S2 Cryo-Lorentz TEM of $Cr_{1/3}TaS_2$. . . . . . . . . . . . . . . . . . . . . . . . . . . 1
S3 SEM-EDX spectra for long- and short-period $Cr_{1/3}NbS_2$ samples. . . . . . . 2
S4 Comparison of SEM-EDX spectra for long- and short-period $Cr_{1/3}NbS_2$ samples. 2
S5 Magnetization behaviour of three slow-cooled $Cr_{1/3}NbS_2$ samples. . . . . . . 3
S6 Magnetization behaviour of four fast-cooled $Cr_{1/3}NbS_2$ samples. . . . . . . . 3
S7 Additional characterization of the deficient $Cr_{0.28}NbS_2$ sample. . . . . . . . . 4
S8 Cryo-LTEM characterization of the deficient $Cr_{0.28}NbS_2$ sample. . . . . . . . 4
S9 Schematic diagram of Lorentz-TEM image contrast. . . . . . . . . . . . . . . . 5
S10 Schematic diagram of Lorentz-TEM image contrast under reversed fields. . . 5
S11 Cryo-LTEM of long-period $Cr_{1/3}NbS_2$ with decreasing fields. . . . . . . . . . 6
S12 Thickness maps of $Cr_{1/3}NbS_2$ S/TEM lamellae. . . . . . . . . . . . . . . . . 6
S13 Analysis of magnetic chirality in the long- and short-period $Cr_{1/3}NbS_2$ samples. 7
S14 Atomic origins of chiral magnetic order. . . . . . . . . . . . . . . . . . . . . . 8
S15 Annihilation of soliton dislocations under applied field. . . . . . . . . . . . . 8
S16 Cryo-LTEM of short-period $Cr_{1/3}NbS_2$ sample for fields ramping up and down. 9
S17 Cryo-LTEM of short-period $Cr_{1/3}NbS_2$ showing abrupt onset of CSL order. . 9

## List of Tables

S1 Elemental concentrations measured by SEM-EDX. . . . . . . . . . . . . . . . . 1

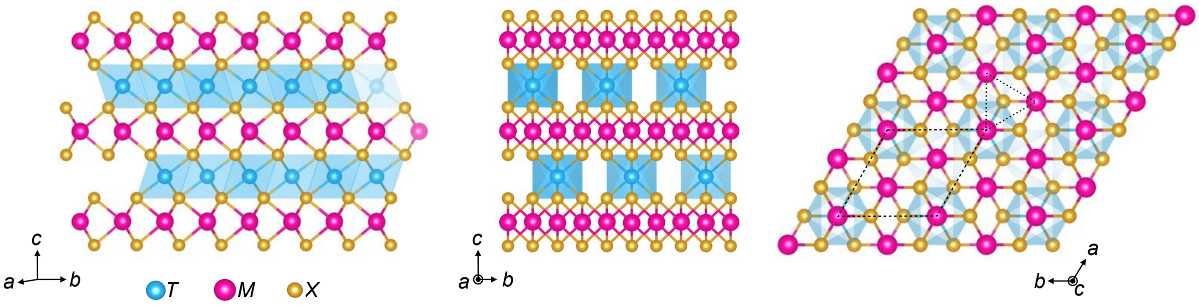


**Figure S1:** Projected crystal views of the $x = 1/3$ intercalated TMD structures, showing intercalants occupying the pseudo-octahedral sites within the van der Waals gap of the host $2H$-TMD lattice. The intercalants order in a $\sqrt{3} \times \sqrt{3}$ superlattice in each plane with the superlattices in consecutive planes along $c$ offset such that intercalants do not occupy the octahedral sites directly above or below each other, reducing the crystalline symmetry from $P6_3/mmc$ to $P6_322$ [1]. The model shown here has right-handed chirality.

| Sample | At%: | Cr | Nb | S | Normalized ratios Cr:Nb:S |
|---|---|---|---|---|---|
| Long-period | | 9.7 | 29.1 | 61.1 | 0.33 : 1 : 2.10 |
| Short-period | | 9.7 | 29.4 | 60.9 | 0.33 : 1 : 2.07 |
| Cr-deficient | | 8.4 | 29.8 | 61.7 | 0.28 : 1 : 2.07 |

Table S1: Atomic percent concentrations of the long- and short-period CSL crystals studied in the main text as well as the Cr-deficient crystal shown in Figure 3c. Concentrations are extracted from the SEM-EDX presented in Figures S3, S4, and S7.

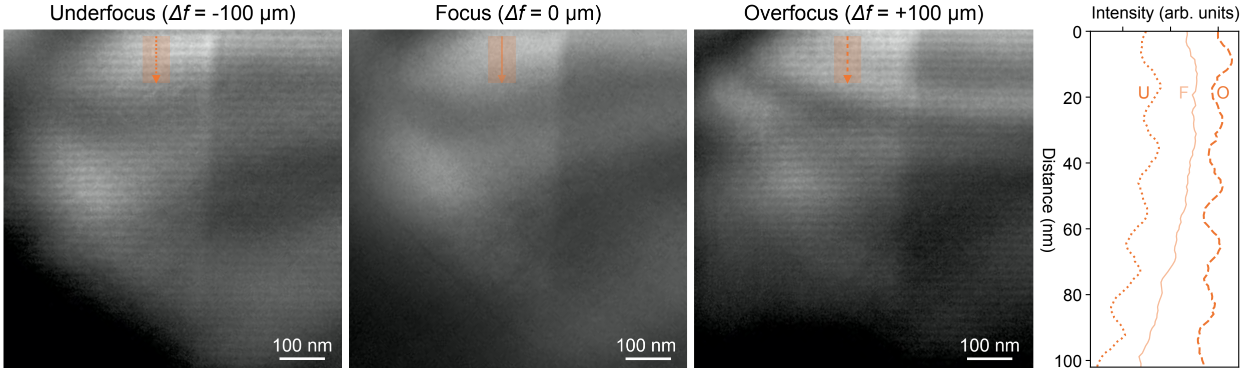


**Figure S2:** Demonstration of Fresnel contrast cryogenic Lorentz TEM imaging of the $Cr_{1/3}TaS_2$ sample near 100 K. At underfocus (negative defocus) conditions (left), the helical magnetic texture is apparent as periodic contrast along the helical axis. In focus (center) the magnetic contrast disappears. At overfocus (positive defocus) conditions (right), the helical texture is again visible with inverted contrast from the underfocus image. Line profiles of the LTEM image intensity over a fixed region marked by the orange arrow are plotted at the far right, with dotted, light, and dashed lines marking the underfocus ("U"), focus ("F"), and overfocus ("O") conditions, respectively.

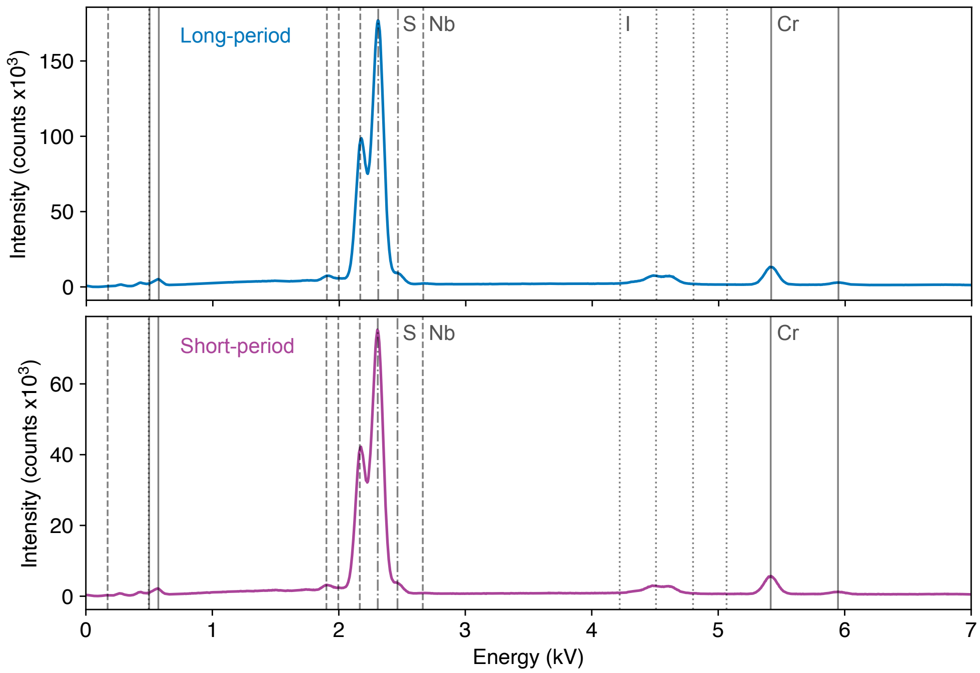


**Figure S3:** Elemental analysis by scanning electron microscopy (SEM) energy dispersive x-ray (EDX) spectroscopy of the long- (top) and short-period (bottom) $Cr_{1/3}NbS_2$ samples. Characteristic peaks associated with Cr, Nb, S, and I (the transport agent) are marked by different line styles.

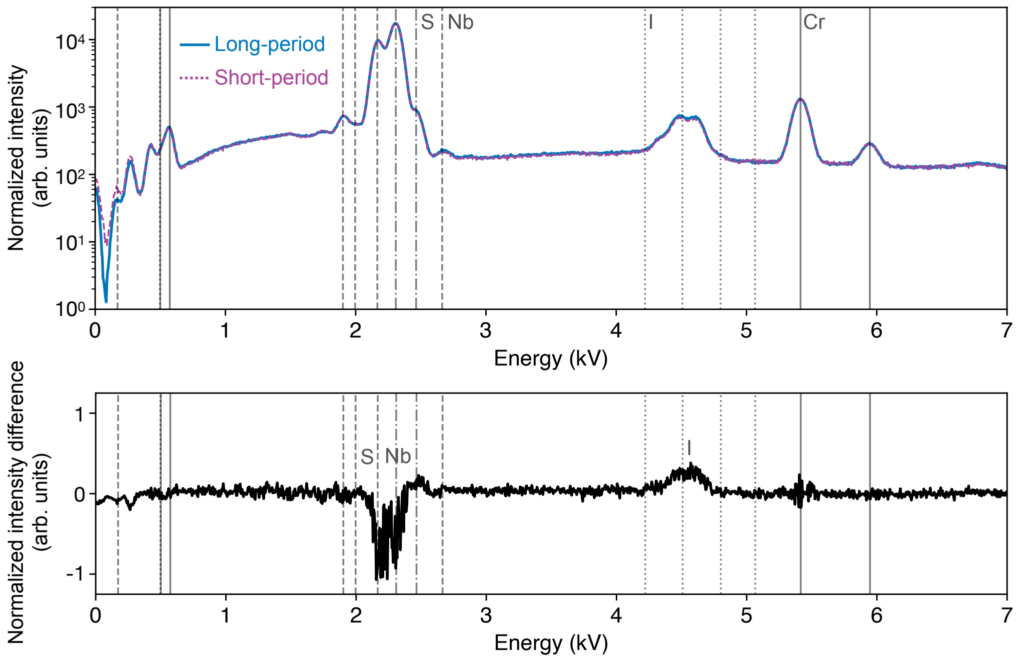


**Figure S4:** (top) Overlaid EDX spectra for the long- and short-period $Cr_{1/3}NbS_2$ samples normalized to the total counts and plotted on a log scale to emphasize differences in the weaker signals. (bottom) Difference in normalized EDX spectra for the long-period minus the short-period $Cr_{1/3}NbS_2$ sample. The main differences in elemental signals are in the TMD species, Nb and S, and in the residual I transport agent. Notably, the difference in Cr signal between the two samples is trivial within the precision of these measurements.

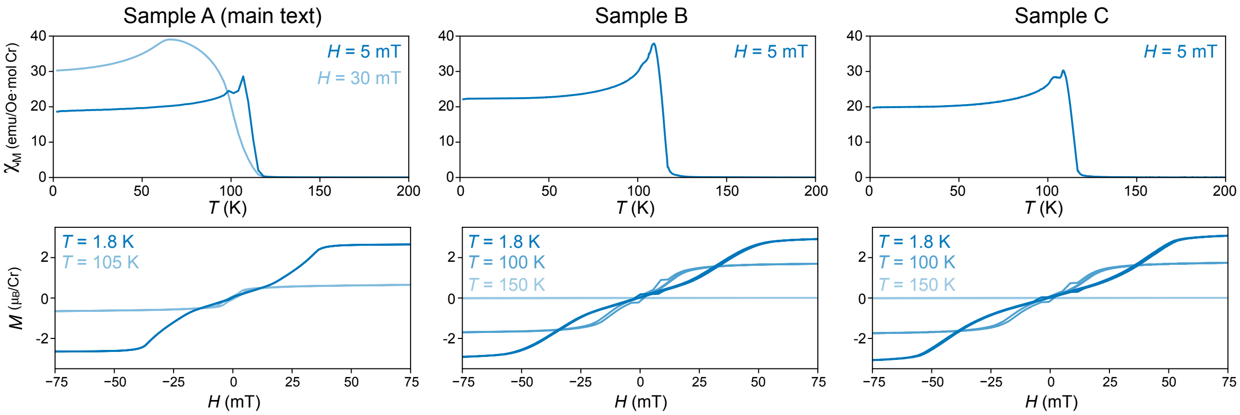


**Figure S5:** Magnetic susceptibility, $\chi_M$, as a function of temperature, $T$, and magnetization, $M$, as a function of magnetic field, $H$, for three $Cr_{1/3}NbS_2$ samples synthesized in the same batch with the slow cooling rate (20 °C/hr). Sample A is the long-period sample studied in the main text.

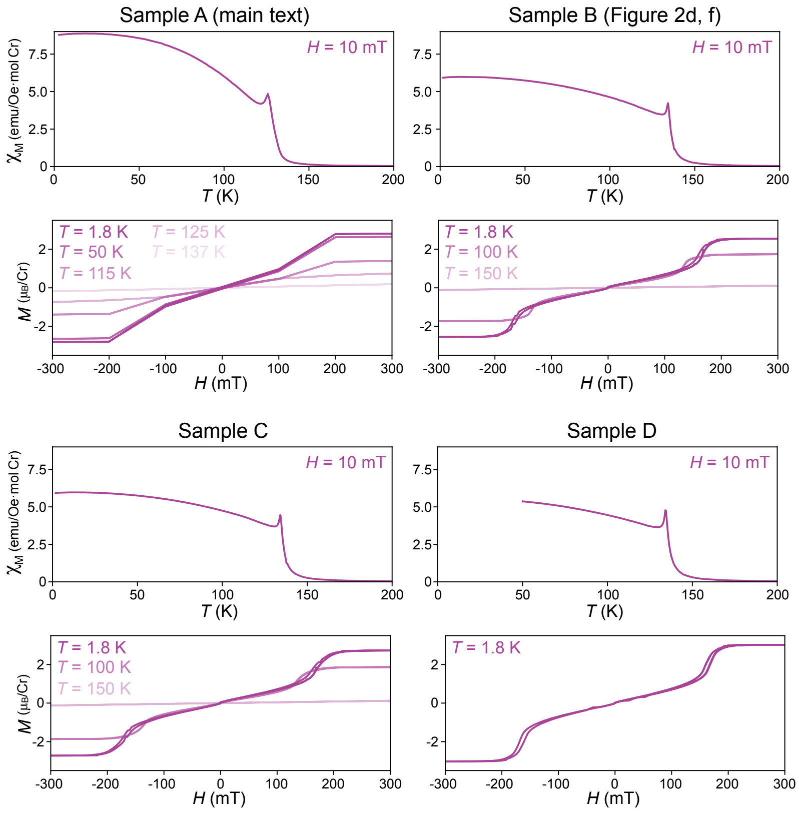


**Figure S6:** Magnetic susceptibility, $\chi_M$, as a function of temperature, $T$, and magnetization, $M$, as a function of magnetic field, $H$, for four $Cr_{1/3}NbS_2$ samples synthesized in the same batch with the fast cooling rate (60 °C/hr). Sample A is the short-period sample studied in the main text. Measurements from Sample B are included in Figure 2d, f of the main text.

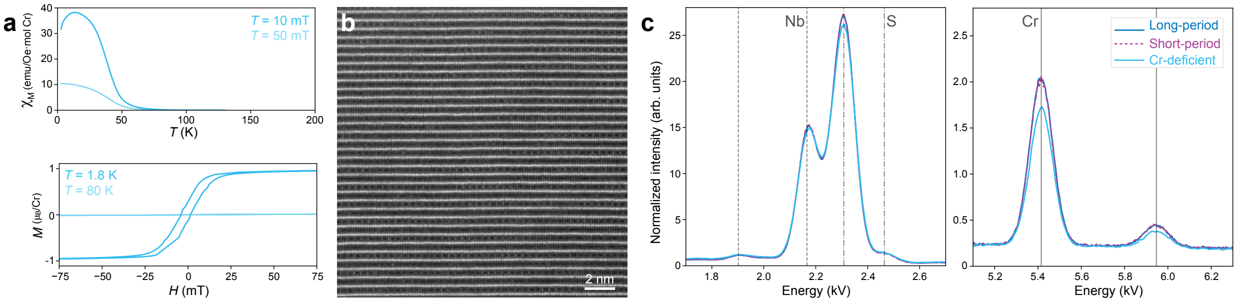


**Figure S7:** Additional characterization of the deficient $Cr_{0.28}NbS_2$ sample shown in Figure 3c of the main text, including **(a)** magnetic susceptibility, $\chi_M$, as a function of temperature, $T$, and magnetization, $M$, as a function of magnetic field, $H$; **(b)** a larger field-of-view HAADF-STEM image showing the density of Cr-vacancy "pockets"; and **(c)** stoichiometric analysis by SEM-EDX. The EDX spectra in c are normalized to the total counts integrated over the Nb-L1, Nb-L$\alpha$, S-K$\alpha$, and S-K$\beta$ peaks marked with dashed and dashed-dotted lines. Extracted atomic percent concentrations for Cr, Nb, and S are given in Table S1.

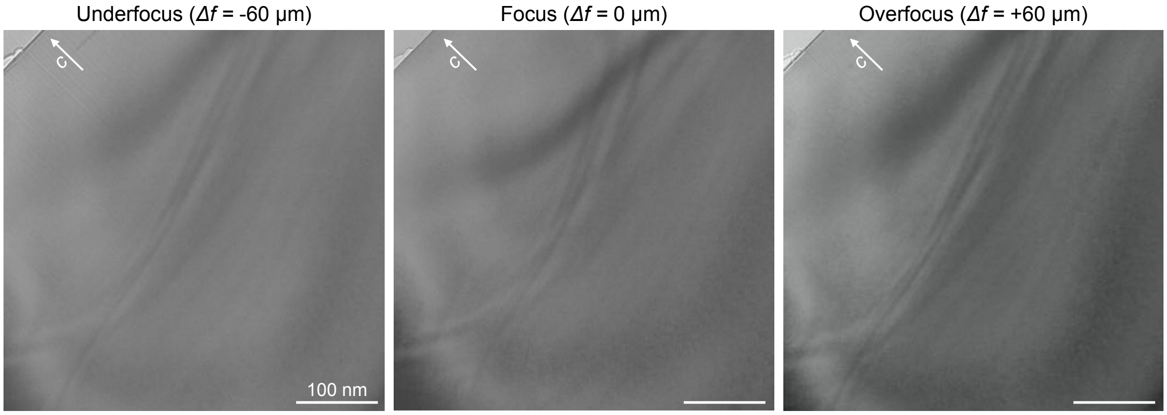


**Figure S8:** Zero-field cryo-LTEM ($T$ ~100 K) investigation of the deficient $Cr_{0.28}NbS_2$ sample shows no signatures of magnetic texture as observed in the $Cr_{0.33}NbS_2$ samples

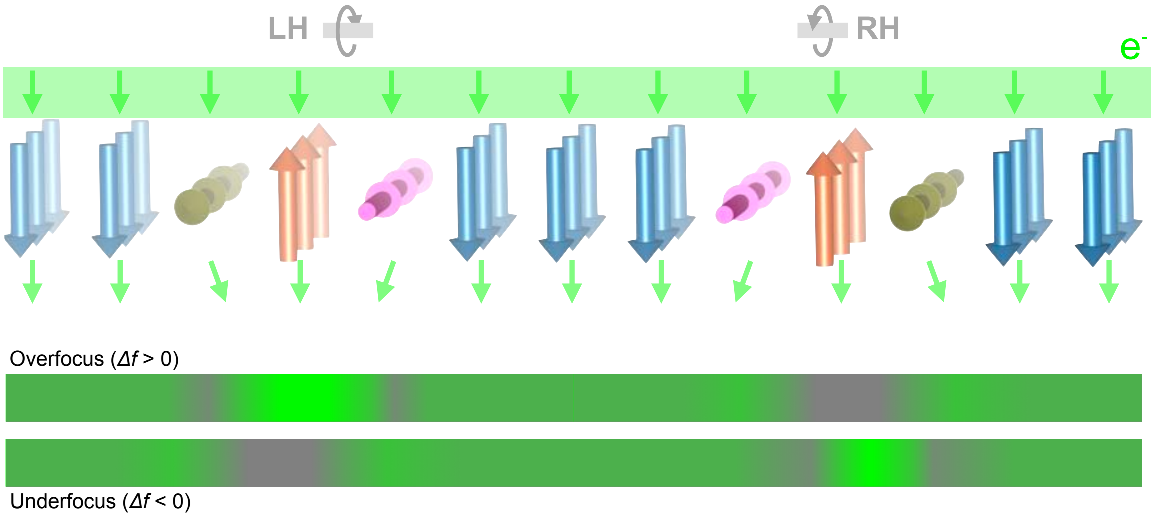


**Figure S9:** Schematic illustration showing the deflection of primary electrons by local moments within the specimen and the expected patterns of bright and dark contrast at soliton walls with opposite chirality in at overfocus (positive defocus) and underfocus (negative defocus) Fresnel Lorentz-TEM (LTEM) imaging conditions.

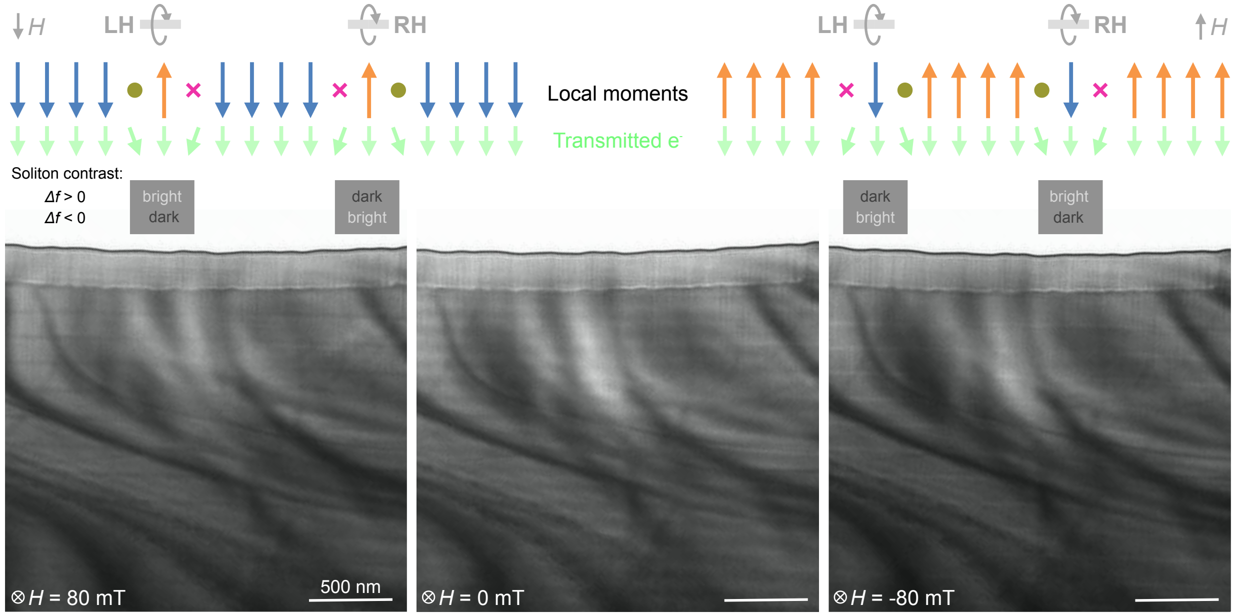


**Figure S10:** Schematic illustration (top) and cryo-LTEM images (bottom) showing the contrast reversal at soliton walls with fixed chirality when the applied field direction is reversed.

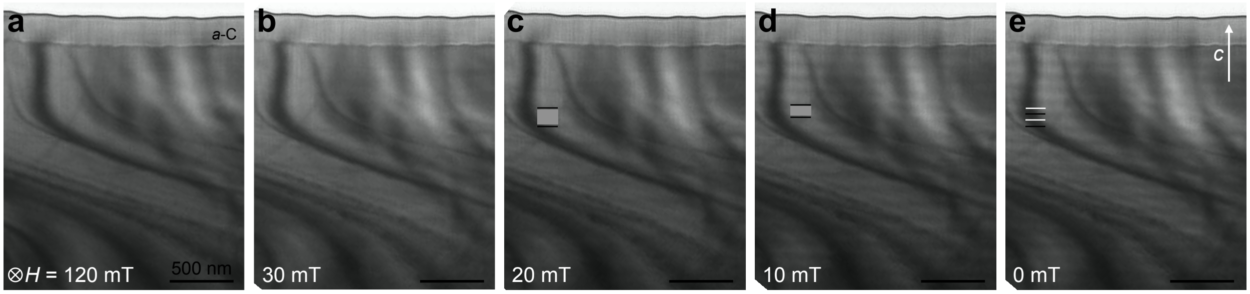


**Figure S11:** Cryo-LTEM images of the long-period sample while ramping the *in situ* field down from above $H_c$, showing hysteresis in the re-emergence of the CSL and HM textures.

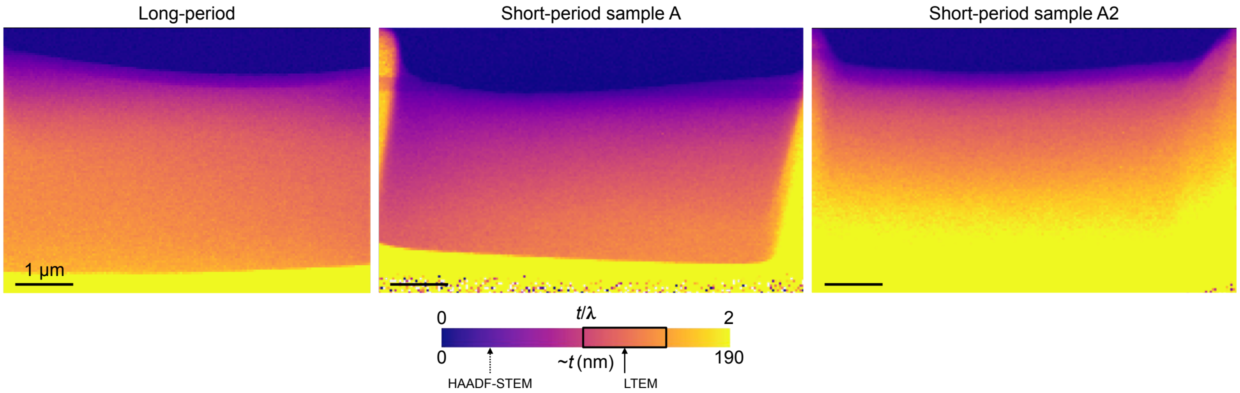


**Figure S12:** Maps of S/TEM lamellae thickness measured by the EELS log-ratio method with incident electron energy of 300 kV. The approximate absolute thickness $\sim t$ is estimated based on an inelastic mean free path $\lambda$ in $Cr_{1/3}NbS_2$ of about 90 nm[2]. Approximate thickness ranges of the regions used for HAADF-STEM and cryo-LTEM imaging are marked.

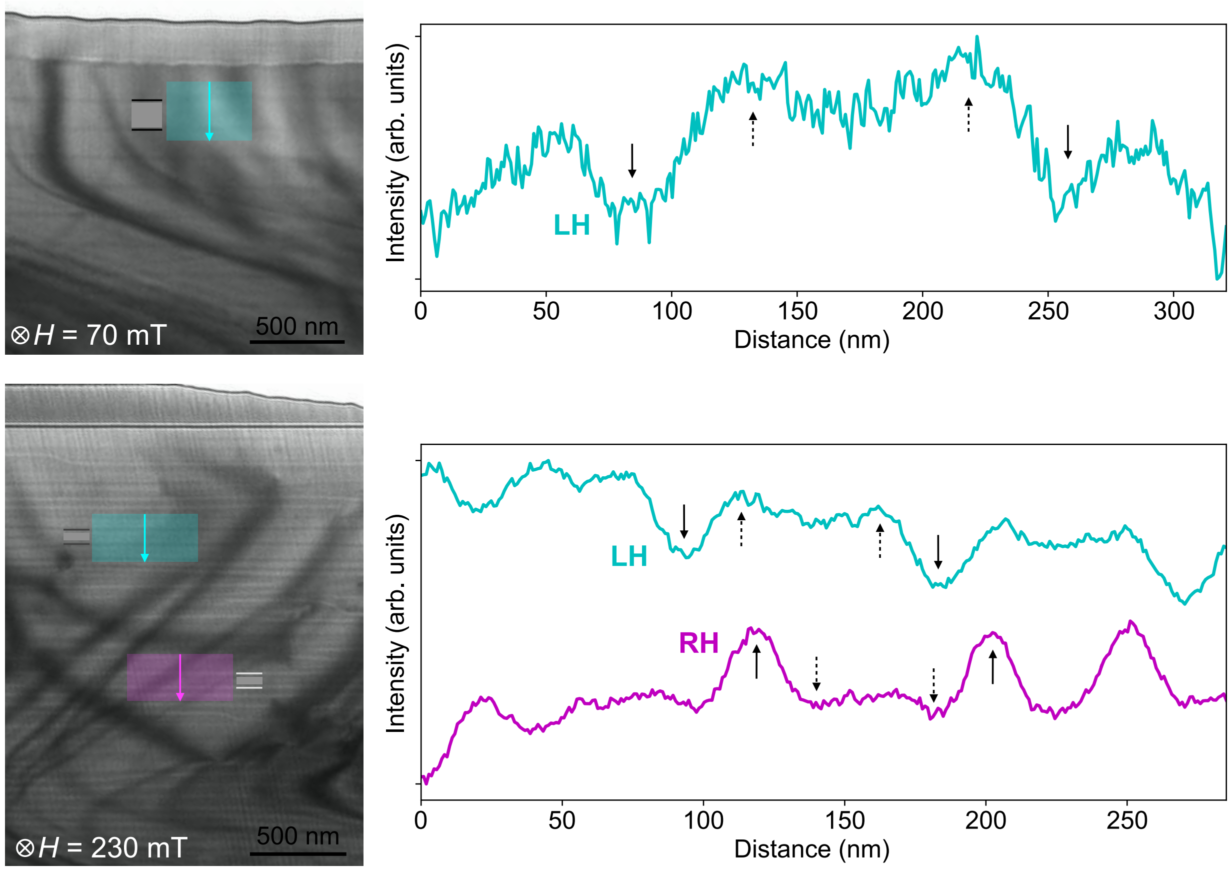


**Figure S13:** Analysis of magnetic chirality in the (top) long-period and (bottom) short-period $Cr_{1/3}NbS_2$ samples based on underfocus ($\Delta f < 0$) cryogenic (T $\sim$ 100 K) LTEM images. Line profiles of image contrast from the marked regions show characteristic contrast for left- (right-) handed soliton walls: notably, strong dips (peaks) in contrast at the center of the solution wall with weaker peaks (dips) in contrast on either side relative to the intermediate contrast observed in the locally ferromagnetic regions[3,4].

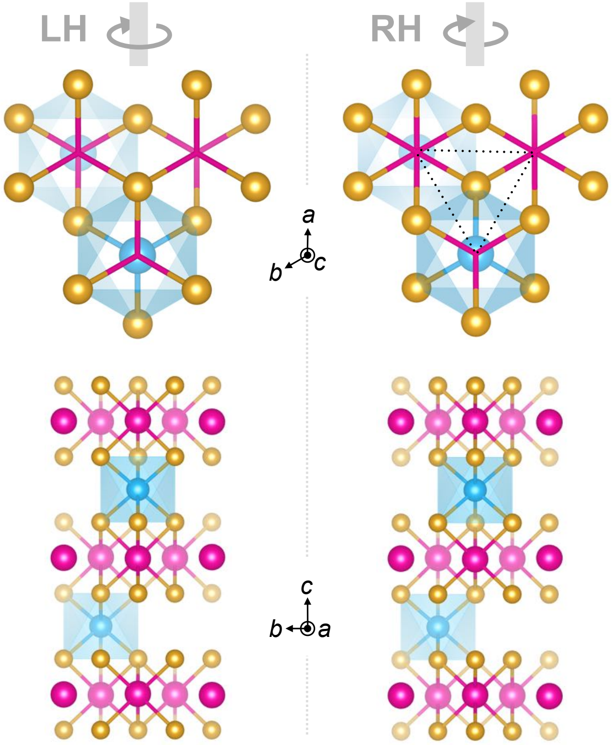


**Figure S14:** Atomic models of left- and right-handed $T_{1/3}MCh_2$ structures. The colors follow the same convention as other models. For clarity, the $M$ atoms are not shown in the $c$-axis view (top panels). The lighter (darker) Cr is intercalated in the bottom (top) van der Waals gap. The dashed triangle marks the same region as that in Figures 1a and S1. In projection imaging of the atomic lattice (i.e., HAADF-STEM), the left- and right-handed structures are indistinguishable.

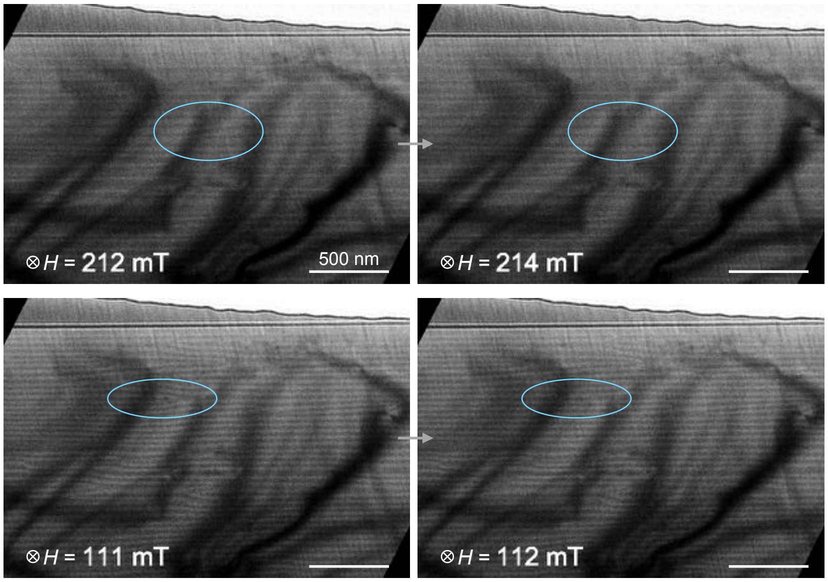


**Figure S15:** Selected still frames from Supplemental Video 2, highlighting two distinct types of soliton dislocation annihilation: (top) two dislocations of opposite sign annihilate as a pair, similar to the description by Dussaux, et al.[5]; (bottom) a single dislocation appears to "self-annihilate" without forming an opposite pair.

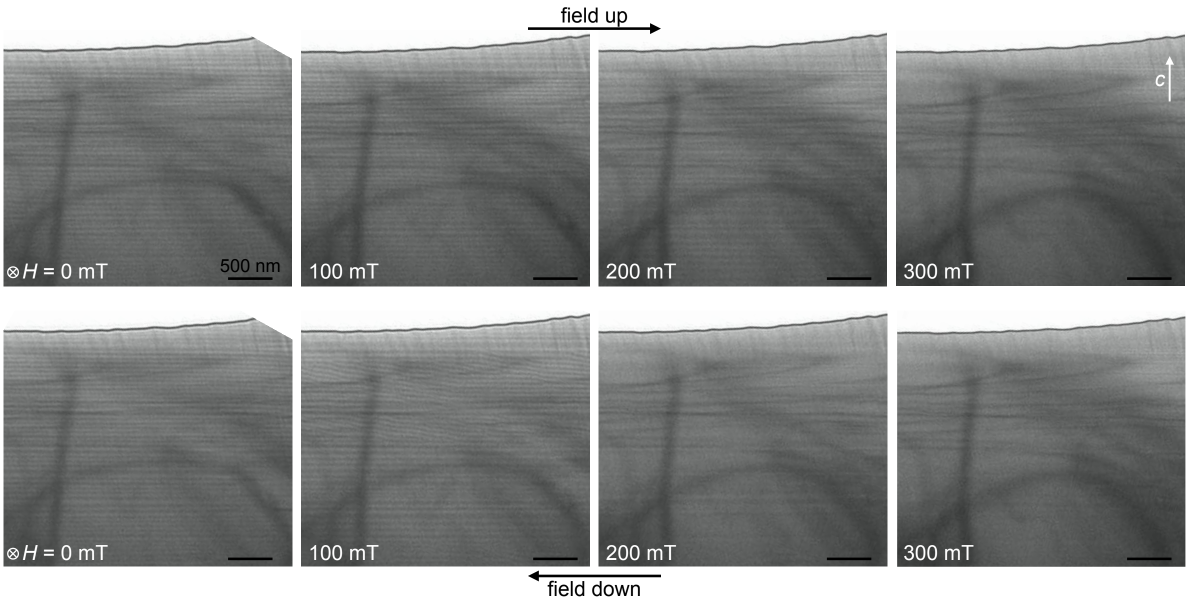


**Figure S16:** Hysteretic behavior in the CSL evolution during external field ramping up (top) and down (bottom) of a second TEM lamella (short-period sample A2 in Figure S12) prepared from the same sample discussed throughout the main text.

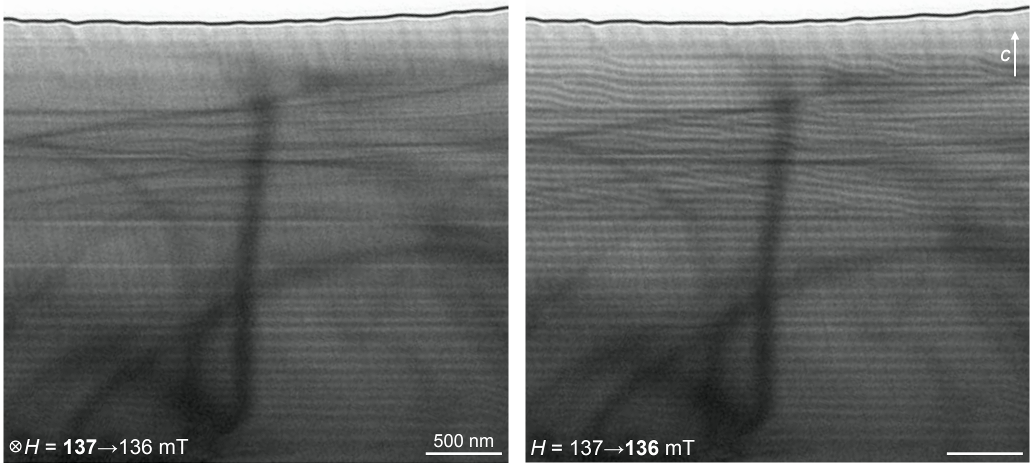


**Figure S17:** Abrupt onset of CSL order upon decreasing external field observed in a second TEM lamella (short-period sample A2 in Figure S12) prepared from the same sample discussed throughout the main text. The change in field strength between image frames is less than 1 mT.

## References


(1) Xie, L. S.; Husremovic, S.; Gonzalez, O.; Craig, I. M.; Bediako, D. K. Structure and Magnetism of Iron-and Chromium-Intercalated Niobium and Tantalum Disulfides. *Journal of the American Chemical Society* **2022**, *144*, 9525–9542.

(2) Egerton, R.; Cheng, S. Measurement of local thickness by electron energy-loss spectroscopy. *Ultramicroscopy* **1987**, *21*, 231–244.

(3) Togawa, Y.; Koyama, T.; Takayanagi, K.; Mori, S.; Kousaka, Y.; Akimitsu, J.; Nishihara, S.; Inoue, K.; Ovchinnikov, A.; Kishine, J.-i. Chiral magnetic soliton lattice on a chiral helimagnet. *Physical Review Letters* **2012**, *108*, 107202.

(4) Togawa, Y.; Koyama, T.; Nishimori, Y.; Matsumoto, Y.; McVitie, S.; McGrouther, D.; Stamps, R.; Kousaka, Y.; Akimitsu, J.; Nishihara, S., et al. Magnetic soliton confinement and discretization effects arising from macroscopic coherence in a chiral spin soliton lattice. *Physical Review B* **2015**, *92*, 220412.

(5) Dussaux, A.; Schönherr, P.; Koumpouras, K.; Chico, J.; Chang, K.; Lorenzelli, L.; Kanazawa, N.; Tokura, Y.; Garst, M.; Bergman, A., et al. Local dynamics of topological magnetic defects in the itinerant helimagnet FeGe. *Nature Communications* **2016**, *7*, 12430.
